# COVID-19 Lockdowns—Effect on Concentration of Pharmaceuticals and Illicit Drugs in Two Major Croatian Rivers

**DOI:** 10.3390/toxics10050241

**Published:** 2022-05-10

**Authors:** Draženka Stipaničev, Siniša Repec, Matej Vucić, Mario Lovrić, Göran Klobučar

**Affiliations:** 1 Croatian Waters, Central Water Management Laboratory, Ulica grada Vukovara 220, 10000 Zagreb, Croatia; drazenka.stipanicev@voda.hr (D.S.); sinisa.repec@voda.hr (S.R.); 2 Department of Biology, Faculty of Science, University of Zagreb, Rooseveltov trg 6, 10000 Zagreb, Croatia; matej.vucic@biol.pmf.hr; 3 Know-Center, Inffeldgasse 13/6, A-8010 Graz, Austria; 4 Centre for Applied Bioanthropology, Institute for Anthropological Research, 10000 Zagreb, Croatia

**Keywords:** pharmaceuticals, illicit drugs, qsar, river water, COVID-19, machine learning, antibiotics, dexamethasone

## Abstract

In order to prevent the spread of COVID-19, contingency measures in the form of lockdowns were implemented all over the world, including in Croatia. The aim of this study was to detect if those severe, imposed restrictions of social interactions reflected on the water quality of rivers receiving wastewaters from urban areas. A total of 18 different pharmaceuticals (PhACs) and illicit drugs (IDrgs), as well as their metabolites, were measured for 16 months (January 2020–April 2021) in 12 different locations at in the Sava and Drava Rivers, Croatia, using UHPLC coupled to LCMS. This period encompassed two major Covid lockdowns (March–May 2020 and October 2020–March 2021). Several PhACs more than halved in river water mass flow during the lockdowns. The results of this study confirm that Covid lockdowns caused lower cumulative concentrations and mass flow of measured PhACs/IDrgs in the Sava and Drava Rivers. This was not influenced by the increased use of drugs for the treatment of the COVID-19, like antibiotics and steroidal anti-inflammatory drugs. The decreases in measured PhACs/IDrgs concentrations and mass flows were more pronounced during the first lockdown, which was stricter than the second.

## 1. Introduction

Coronavirus disease 2019 (COVID-19) caused by severe acute respiratory syndrome coronavirus 2 (SARS-CoV-2) has created one of the greatest global health and socioeconomic crises in recent history [1,2]. A wide range of public health and social measures were implemented worldwide to prevent the spreading of COVID-19 and reduction of its burden on the health system [3]. By the end of March 2020, half of the world population was under some form of lockdown [4]. These contingency measures also had some positive effects on the environment, particularly improvement in air quality and reduction in water and noise pollution [1,2,5]. So far, most of the published data regarding improvement of environmental conditions had been on air quality, followed by several studies describing reduction of river pollution, mainly in India [6,7,8,9], and mainly regarding organic pollution (pH, BOD, nitrates, and nitrites) or metals. These effects are the result of the seizure of industrial activities and tourism [10]. However, in most countries affected by the COVID-19 pandemic, industrial activities were not halted. Furthermore, several studies detected increased concentrations of COVID-19 related pharmaceuticals in the aquatic environment and sewage sludge during pandemic [11,12]. Nevertheless, it can be assumed that the severe imposed restrictions of social interactions also reflected on the water quality of rivers receiving wastewaters from urban areas such as Zagreb, the capital and largest city (approx. 1 million inhabitants) of Croatia.

**Figure 1:**
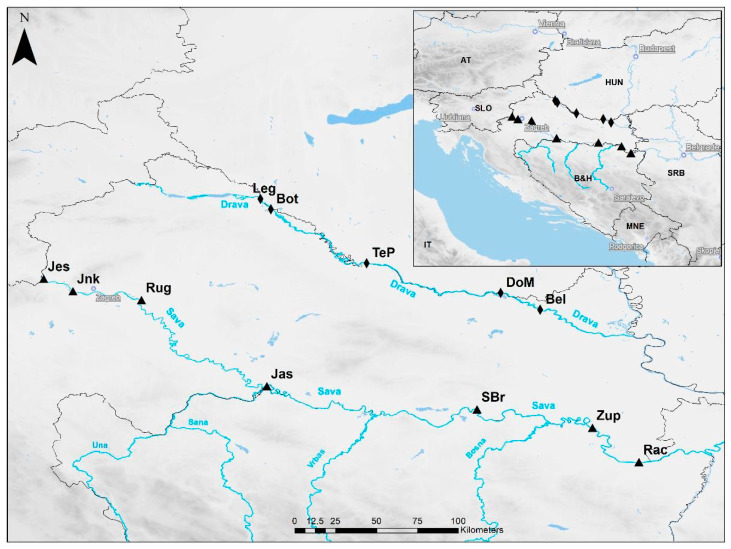
A map of Croatia with the collection sites of water samples for analysis of PhACs.

In Croatia, the Sava and Drava rivers are the two longest rivers and main recipients of wastewaters from urban and rural areas, and are major tributaries of Danube River. Both rivers are transboundary, and the majority of their course is on the border with neighboring countries. The Sava River basin is the largest river basin in Southeast Europe with a catchment area of 97,700 km^2^ and population nearing 8,000,000. It is the third longest tributary, and the largest by discharge, of the Danube River. It runs through four countries: Slovenia, Croatia, Bosnia and Herzegovina, and Serbia (Figure 1). The lower 304 km out of 510 km of the Croatian part of the Sava River basin is on the border between Republic of Croatia and Bosnia and Hercegovina. The Drava River has a 40,150 km² catchment; it is the fourth longest tributary of the Danube River that runs from Italy, through Austria and Slovenia to Croatia, where it forms the border between Croatia and Slovenia, and Croatia and Hungary. Out of a total of 323 km of the Croatian part of the Drava River basin, 20 km belongs to border with Slovenia and 136 km with Hungary.

The epidemic of COVID-19 disease in the Republic of Croatia was announced on 11 March 2020 (https://vlada.gov.hr/coronavirus-protection-measures/28950; accessed on 5 June 2021). On the same day, the World Health Organization (WHO) declared the worldwide pandemic of COVID-19 [13]. On 19 March 2020 Croatia closed all non-essential activities—shopping centers, bars and coffee shops, restaurants, cinemas, theatres, reading rooms, libraries, gyms, sports centers and competitions, fitness centers, recreation centers, dance schools, children’s and other workshops, religious gatherings, exhibitions, fairs, and nightclubs and discos (https://askabout.io/covid-19/ask/what-is-the-government-response-timeline-for-croatia/; accessed on 8 June 2021). Travelling bans and restrictions, as well as closing of schools, followed shortly after. Private gatherings of more than five people were also banned. These measures were relaxed between 27 April and 11 May 2020 when the bans were lifted. Slovenia started a similar set of restrictions in the second half of the March 2020 that lasted until the start of May (https://askabout.io/covid-19/ask/what-is-the-government-response-timeline-for-slovenia/; accessed on 9 June 2021). In Bosnia and Hercegovina (B&H), the state of emergency was declared in late March 2020 and was lifted in late May same year. In Hungary, the first lockdown was imposed between 11 March and 4 May. A second set of major restrictions (second lockdown), following an increased number of COVID-19 patients in Croatia during fall and winter 2020, were announced on 28 November 2020. This time, the restrictions were less severe than in the first lockdown and included locking down cafés and restaurants, and a ban on wedding parties, fairs, and most sports events. These restrictions lasted until 15 February, after which the restrictions were gradually lifted, starting with opening of cafés terraces from 1 March. Again, a similar time scale was applied in Slovenia with closure of restaurants, bars and coffee shops, hairdressers, barbers, cosmetic salons, fitness gyms, and hotels from 24 October 2020 and lasting almost six months (until April 2021). In B&H, COVID-19 measures were initiated across the country on October 26 and were much lighter than the first lockdown (most businesses, shops, and restaurants remained open). These measures gradually came to an end in April. In Hungary, a second lockdown started mid-November 2020 and lasted until February 2021.

Pharmaceutically-active compounds and their metabolites (PhACs) represent a variety of man-made organic chemicals with therapeutic properties and uses that are considered as emerging contaminants (ECs). Rivers receive pharmaceuticals from raw or treated municipal and hospital waste/wastewaters, pharmaceutical manufacturing, and animal farming [14,15,16,17]. Concentrations of PhACs in surface waters range from very low ng/L to low µg/L and up to mg/L (e.g., near PhACs manufacturing facilities) [18,19]. Besides PhACs, another group of ECs, illicit drugs and their metabolites (IDrgs), is becoming more present in the freshwater ecosystems [20]. Due to their poor or incomplete removal by wastewater treatment plants (WWTPs), different IDrgs have been detected in raw or treated wastewater, in surface water, and tap water (ng/L to mg/L) [15,16,17,21].

The aim of our work was to investigate possible variations in concentrations of pharmaceuticals and illicit drugs, as well as their metabolites (PhACs/IDrgs), in the Rivers Sava and Drava during the 16-month time period that included two periods of lockdown (March–May 2020 and November 2020–March 2021) in Croatia and bordering countries. Apart from the usage of PhACs/IDrgs in the general population, their concentration in the river water can be influenced by the present quantity of river water, i.e., the river flow (m^3^/s) that is mainly the result of precipitations and their seasonality. In order to exclude the possible influence of the variations in the river flow that could have affected the concentrations of the measured PhACs/IDrgs in the river water, we have expressed our results as mass flow over time and sites.

## 2. Materials and Methods

### 2.1. Site Description and Sampling

Each month from January 2020 to April 2021, surface water samples were collected from seven sites along the Sava River: Jesenice (Jes; 45.860595 N, 15.692357 E), Jankomir (Jnk; 15.692357 N, 15.852741 E), Rugvica (Rug; 45.743684 N, 16.228377 E), Jasenovac (Jas; 45.268587 N, 16.915962 E), Slavonski Brod (SBr; 45.141268 N, 18.070956 E), Županja (Zup; 45.039882 N, 18.703391 E), and Račinovci (Rac; 44.851851 N, 18.959196); and from five sites along the Drava River: Legrad (Leg; 46.297943 N, 16.880991 E), Botovo (Bot; 46.241223 N, 16.937914 E), Terezino Polje (TeP; 45.944342 N, 17.462874 E), Donji Miholjac (DoM; 45.782751 N, 18.20062 E), and Belišće (Bel; 45.69049 N, 18.416862 E) (Figure 1).

We collected water samples in sterile polycarbonate flasks (250 mL) as described in Stipaničev et al. [22], and transported them at 4 °C to be analyzed the next day. Prior to analyses, water samples were centrifugated (10,000 rpm for 20 min) and supernatants were used for the analyses. The use of on-line solid phase extraction (SPE) requires that there be no suspended matter in the water samples. The centrifuging is efficient enough to eliminate any suspended matter that might alter the chromatography system. Filtration was, therefore, not necessary [23]. Ultrapure laboratory water samples were always processed in parallel with the environmental water samples. The data for water concentration of the contaminants are given in ng/L (Table S1, Supplementary Material). The obtained river flow data are in m^3^/s (Table S2, provided by Croatian Meteorological and Hydrological Service). For the purpose of this work, we have calculated the contaminant mass load per site and river in mg/s, i.e., the total mass load of the contaminant passing a measurement station in the river per second.

### 2.2. Reagents and Chemicals

Acetonitrile, methanol, isopropanole, water, ammonium formate, and formic acid, all of LC/MS grade, were purchased from J.T. Baker (Fisher Scientific, Pittsburgh, PA, USA). Forensic Toxicology Comprehensive Mix, Submix 1-10 C analytical standards (purity > 98%) were purchased from Agilent Technologies (Santa Clara, CA, USA). Azithromycin, 4-acetamidoantipyrine, 4-formylaminoantipyrine, bisoprolol, metoprolol, torsemide, and total THC—Calibration Mix HPLC 2 were purchased from Absolute Standards (Hamden, CT, USA) and vet drugs kit 101 components from Labinstruments (Castellana Grotte, Italy). Individual solutions of 100 µg/mL were prepared in methanol for all standards and were kept in the dark at −18 °C. Standard mixtures of 10 µg/L for each compound were prepared in methanol. Working solutions of 1 µg/L were prepared in water and from that solution seven-point calibration curves were generated.

### 2.3. Method Performance-Online SPE-LC-MS/MS

The online SPE systems present the advantage to provide automatic and efficient sample loading, clean-up, desorption, separation, and detection at the same time, to reduce the sample volume, save time and solvents, prevent sample contamination and PPCP loss, and improve the method performance. The reduction in sample volume that could decrease sensitivity is normally compensated for by the increase in sensitivity as all the analyte retained in the sorbent passes to the chromatographic column [24].

The online SPE–UHPLC-MS/MS method was validated in accordance with the ISO/IEC 17025 guideline [25]. The parameters evaluated in the validation process were specificity, linearity, LOD and LOQ, accuracy, and precision. The specificity of the method was established by comparing the control sample, spiked sample, and real sample chromatogram. No unwanted peak was observed at the retention time of analyte peak, which determine the specificity of the method. Calibration curves were built as a function of the analyte concentration. The method linearity was constructed by a seven-point calibration curve of PPCPs in water in the concentration range of 0.001–0.500 µg/L using the linear least square method. Linear response was observed in all cases (R^2^ > 0.998) for all the selected PPCPs. Calibration standards were extracted and treated by the online SPE procedure in exactly the same manner as the environmental samples [26]. Ultrapure laboratory water samples were always processed in parallel with the environmental water samples. LODs and LOQs were determined as the minimum amount detectable of analyte with a signal-to-noise ratio of 3 and 10 and theoretical limit of quantification (LOQ) ranged from 0.2–0.9 ng/L. Data processing was performed using LabSolutions Insight software (Shimadzu Corporation, Japan), with no manual correction. Accuracy and precision of the measurements are controlled after each twenty-samples series by injection of quality control samples at low level (limit of quantification) and intermediate level (50 ng/L). Acceptance criteria for accuracy were recoveries between 70% and 125% and for repeatability relative standard deviations lower than 15%.

### 2.4. Instrumentation and Analysis

A Nexera X2 UHPLC was coupled to LCMS-8060 high sensitivity triple quadrupole (Shimadzu Corporation, Kyoto, Japan). UHPLC analysis consists of a 33 min gradient using water/MeOH/acetonitrile mobile phases with ammonium formate and formic acid as additives to enhance ionization and improve chromatography in positive and negative ESI mode. The online SPE allows a large injection volume (1000 µL) where the sample passes through the SPE column Evolute Express ABN 20 µm, 30 × 2.1 mm (Biotage, Uppsala, Sweden) with ultrapure water loading. After all analytes of interest are retained on the SPE by combining the opening and closing of the valve at a certain point, the combination of mobile phases moves in the opposite direction from the elution and in gradient mode begins to wash the analytes of interest from the SPE column to the analytical column. Separation was performed on an ACQUITY UPLC HSS T3 Column, 100 Å, 1.8 µm, 2.1 mm × 100 mm (Waters Corp., Milford, MA, USA). The column chamber was tempered to 40 °C. In the positive and negative ionization mode, the mobile phases are composed of solvent A (10 mM ammonium formate/water/0.1% formic acid) and solvent B (10 mM ammonium formate/methanol/acetonitrile 50:50/0.1% formic acid). Mass spectrometry was performed with a triple quadrupole fitted with an ESI interface and controlled by LabSolution software. Typical interface conditions were optimized for maximum intensity of the precursor ions as follows: nebulizing gas 3 L/min, heating gas 10 L/min, drying gas 10 L/min, interface temperature 300 °C, desolvation line 150 °C, and heating block 300 °C. The ESI polarity ionization mode was set individually for each target compound. Positive and negative polarity modes were used simultaneously during the same analytical run. MRM transitions were selected and tuned individually for each analyte. To optimize the mass spectrometer, a 500 μg/L standard solution of each analyte was infused directly. The specific and intense product ions of each target analyte were used for quantification, and two products ions were used as a qualifier ions for confirmatory purposes.

### 2.5. Measured Pharmaceuticals and Illicit Drugs

Eighteen representative PhACs and IDrgs (15 and 3, respectively) were analyzed. Seven PhACs were analyzed in Sava and Drava River: stimulants caffeine and cotinine (metabolite of nicotine), anticonvulsants carbamazepine and lamotrigine, 10-hydroxycarbazepine (metabolite of oxcarbazepine), and antidepressant venlafaxine and its metabolite O-desmethylvenlafaxine. An additional 9 PhACs, antibiotic azithromycin; diuretic torasemide; beta-blockers bisoprolol and metoprolol; anti-inflammatory drugs like corticosteroid dexamethasone, 4-acetylaminoantipyrine (metabolite of aminopyrine), and 4-formylaminoantipyrine (metabolite of aminophenazone); neuroleptic amisulpride; and illicit drugs, MDMA-3,4, and cocaine and its metabolite benzoylecgonine, were analyzed in Sava River, making a total of 18 chemicals (Table S1). The listed contaminants were furthermore categorized in eight categories: Antibiotics (ATB), Antidepressants (ATD), Antiepileptics/Neuroleptics (ATE), Illicit Drugs (IDRG), Cardiovascular medicals (CAR), Diuretics (DIUR), Stimulants (STM), and Anti-inflammatory drugs (AID), as shown in Table 1.

### 2.6. Data Processing and Analysis

To understand the difference in the obtained results between the sites, we analyzed the time series data for common patterns. For this purpose, we aggregated the mass flows across the sites (sum over site per time point) where the aggregated data present contaminants total mass flow over time and site. The data were then subjected to principal component analysis (PCA) so we could observe patterns in a lower dimensional space.

To understand how groups of contaminants were utilized during and post-lockdown we have summed their mass flows per category and across the sites in both rivers to obtain mass flow of the eight categories in time.

Data processing and analysis was conducted in Python (v3.7.10, www.python.org; accessed on 15 February 2022) and DataSpell (v2021.3, https://www.jetbrains.com/dataspell/; accessed on 22 February 2022). For statistical modelling and data manipulation, the following libraries were utilized: scikit-learn [27] and pandas [28]. To generate visualizations, we utilized matplotlib [29] and seaborn [30].

### 2.7. Concentration Comparison of Pre- and Post-Lockdown

Differences in pre- and post-lockdown vs lockdown concentrations (Table S1) are evaluated by comparing average contaminant mass flow in times of both lockdowns (mid-March–mid-May 2020 and late October 2020–March 2021) to “normal” periods, i.e., out-of-lockdown (*OOL*). For each PhACs/IDrgs at each measurement site (*i*), the average contaminant mass flow (*L_i_*) for the lockdown periods and the average contaminant mass flow for the *OOL* period were calculated. Then, the *OOL* average contaminant mass flows were divided by the lockdown and are defined here as factor (*F*) for a PhAC/IDrg by Equation (1). The respective standard deviation was also calculated. Both results are presented in Table 2 and Table 3. *F* represents therefore the average ratio for a PhAC in *OOL* vs. *LCK* periods.

(1) $$F=\overset{N}{\underset{i}{\sum }}\frac{\frac{L_{i}\left(OOL\right)}{L_{i}\left(LCK\right)}}{N}\;$$

Data processing and analysis was conducted in Python 3.7 (www.python.org; accessed on 15 February 2022). We replaced the missing values (not quantified) with 1/10 of the respective LOQ value (limit of quantification).

## 3. Results

### 3.1. PhACs/IDrgs Mass Flow Comparison of Pre- and Post-Lockdown

**Figure 2:**
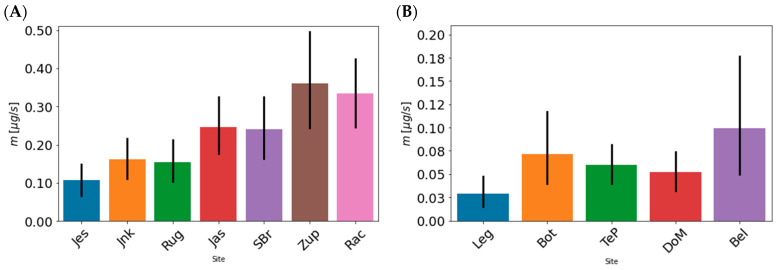

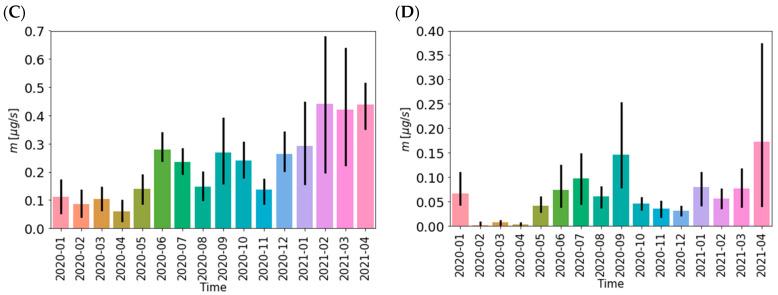
Average PhACs/IDrgs mass flow over the analyzed period of 16 months across all sites in (**A**) Sava River and (**B**) Drava River. Average PhACs/IDrgs mass flow over time and sites for (**C**) Sava River and (**D**) Drava River.

**Figure 3:**
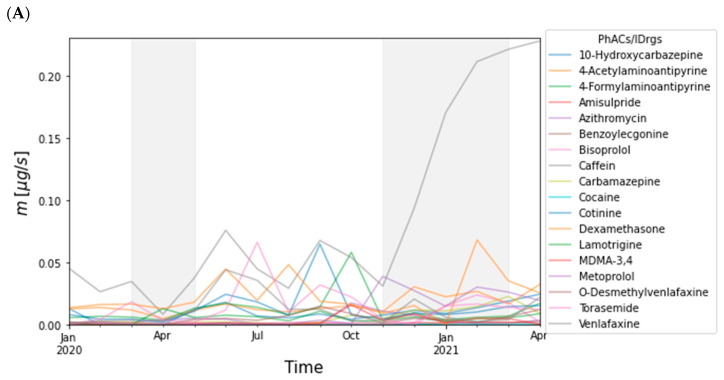

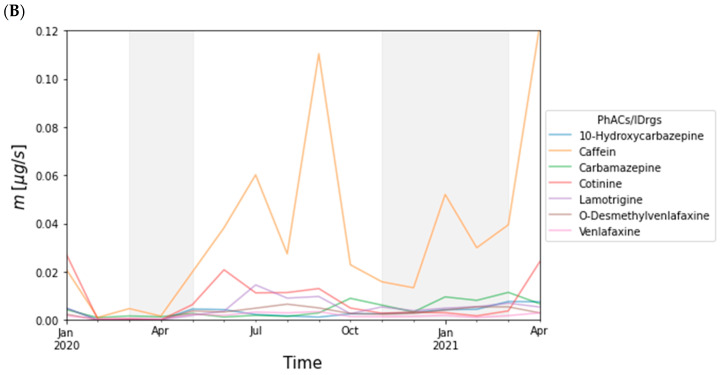
Average mass flow (µg/s) of all measured PhACs/IDrgs across all sites in the rivers during 16 months: (**A**) Sava and (**B**) Drava. Time periods for the first and the second lockdown are marked in grey.

**Figure 4:**
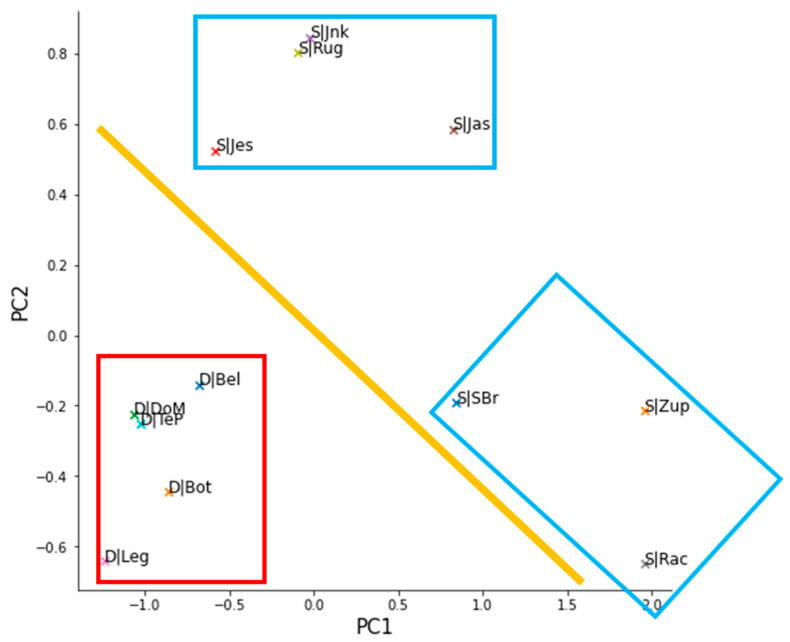
Summed PhACs/IDrgs mass flow over across the sites transformed by principal component analysis (PCA). The plot is showing PCA scores of the sites in the Sava River: Jankomir–S|Jnk, Rugvica–S|Rug, Jasenovac–S|Jas, Jesenice–S|Jes, Županja–S|Zup, Slavonski Brod–S|SBr, Račinovci–S|Rac; and the Drava River: ‘Legrad–D|Leg, Botovo-Oriloš–D|Bot, Terezino Polje-Barc–D|TeP, Donji Miholjac-Dravasabolc–D|DoM, Belišće–D|Bel. The yellow line represents a split between sites in the Sava river and the sites in the Drava river. The blue rectangles represent two assigned groups of the clustered Sava sites, while the red one shows all plotted Drava sites.

**Figure 5:**
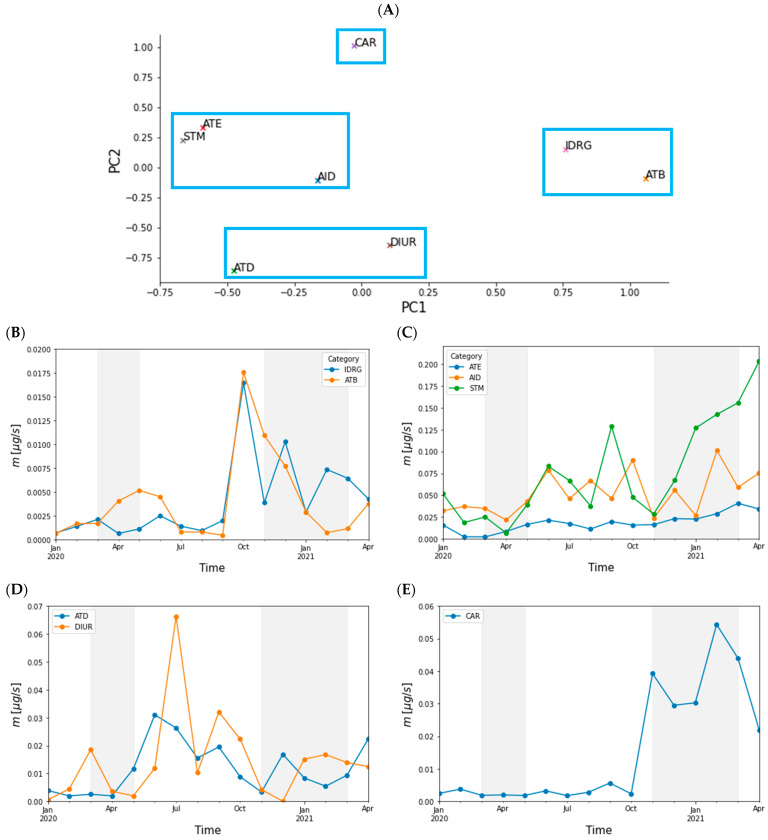
(**A**) PCA scores plot of the summed PhACs/IDrgs grouped by category; (**B**–**E**) from the PCA recovered categories (blue rectangles) are plotted as time series according to their position in the PCA, i.e., similarity of the time-based patterns. The grey areas represent periods of lockdowns.

We inspected the pattern by means of contaminant mass flows per site considering the concentration (Table S1) and water flow (Table S2) at the time of sampling (see Section 4). Mass flows per location and time patterns of total mass flows are presented in Figure 2. It is evident that the mass flows of detected PhACs/IDrgs are continuously increased at downstream sites of the Sava River unlike in Drava River (Figure 2A,B). Lower concentrations and contaminant mass flow in the Drava River are the result of lower water flow and measurement of only 7 PhACs while in the Sava River 17 PhACs/IDrgs were measured. Nevertheless, the average load of PhACs/IDrgs over 16 months during 2020 and 2021, encompassing two periods of Covid lockdown (mid-March–mid-May 2020 and late October 2020–March 2021), showed that, during the lockdowns, there was a decrease in the concentrations and mass flows of measured PhACs/IDrgs in both rivers (Figure 2, Figure 3, Figure 4 and Figure 5, Figures S1 and S2, and Tables S1 and S2). However, it is evident that the first lockdown produced a more prominent decrease in the concentrations and mass flows of the measured PhACs/IDrgs than the second one (Figure 2C,D, Figures S1 and S2).

Mass flow of each PhACs/IDrgs measured in the Sava and Drava Rivers were inspected visually by means of average mass flow over time (Figure 3). The mass flows across all sites and specific PhACs/IDrgs are plotted over time for the two rivers.

Results for the Sava River (Table 2) show that several PhACs have shown a substantial loss in river water mass flows during lockdowns: venlafaxine and o-desmethylvenlafaxine (ATD), 4-formylaminoantipyrine (AID), cotinine (STM), torasemide (DIUR), and dexamethasone (AID). In the Drava River such a significant decrease of mass flows during lockdowns was noted for stimulants cotinine and caffein, as well as lamotrigine (ATE) and venlafaxine (ATD) (Table 3).

### 3.2. Site Comparison

The PCA scores are plotted in Figure 4. The two principal components (PC1 and PC2) in the plot explain 85.5% of the variance in the data and are used here for visualization purposes. From the scores plots, it can be observed that the two rivers, Drava (D) and Sava (S), with their respective sites, are split along the diagonal which is a linear combination of the two components. Besides the clear river split, one can also observe that there are roughly two groups of sites in the Sava River–locations in the upper Sava (Jankomir–S|Jnk, Rugvica–S|Rug, Jasenovac–S|Jas, Jesenice–S|Jes) with lower water flows and locations in the lower part of the river which are affected by the in-flow from southern tributaries from Bosnia and Herzegovina (mainly Una, Vrbas and Bosna rivers) with larger water flows (Županja–S|Zup, Slavonski Brod–S|SBr, Račinovci–S|Rac).

### 3.3. Category Comparison

The group division for the measured PhACs/IDrgs is given in Table 1. The PCA scores plot is shown in Figure 5A. It reveals several clusters and individual groups of PhACs/IDrgs which were plotted together in Figure 5B–E. One such cluster (group 1) is formed by group Antibiotics and Illicit drugs showing a common pattern characterized by an increase of their mass flow after the first lockdown and prior to the second lockdown (Figure 5B). Another cluster (group 2, Figure 5C) consists of groups Antiepileptics/Neuroleptics, Anti-inflammatory drugs, and Stimulants (caffeine and cotinine). It shows a similar pattern to group 1 and appears to be affected by the lockdowns (especially the first one). The third cluster (Figure 5D) shows generally higher values between the lockdowns and consists of Antidepressants and Diuretics. This cluster is more affected by the lockdown than the first two groups, their mass flows are low during lockdowns and increase after them. A further separated PhAC group is Cardiovascular medicals which have an increase in winter times (second lockdown).

## 4. Discussion

The results have clearly shown that the concentrations and cumulative mass flows of measured PhACs/IDrgs in both rivers, Sava and Drava, dropped during the two Covid lockdowns in 2020 and 2021. It is important to stress that the second lockdown (November 2020–February 2021) was less strict and lasted longer than the first one (March 2020–May 2020). Consequently, the decrease in measured total mass flow was less pronounced during the second lockdown.

Both rivers are transboundary in the majority of their flow and are also polluted by countries other than Croatia. Whilst Hungary’s contribution to the water mass of Drava River is modest, the situation with Sava is different and rivers from B&H are contributing significantly to the water mass and flow and, presumably, to the cumulative contamination load of the river. This is also visible in PCA scores of the sites in the Sava River where there is a clear distinction between the upper (Jnk, Rug, Jas, and Jes) and lower part (Zup, SBr, and Rac) of the Sava River. Therefore, it is important to consider the influence of major B&H rivers (Una, Vrbas, and Bosna) on the type and amount of contamination in the Sava River. Furthermore, it is important to emphasize that the lockdowns in Bosnia and Herzegovina were less strict than in Croatia (especially the second one) which probably influenced the cumulative mass flows of the measured PhACs/IDrgs during the lockdown periods in the Sava River in this study. Therefore, the decrease in contamination during the second lockdown is more clearly visible in the Drava River.

Analyses further indicated several PhACs across all sites in the rivers whose concentrations and mass flows were significantly lower during the lockdowns. As lockdowns closed all non-essential activities, including bars and coffee shops, restaurants, and nightclubs and discos, a significant drop in mass flows of caffeine and nicotine metabolite cotinine in river water was expected. Interestingly, antidepressants venlafaxine and o-desmethylvenlafaxine also showed notable decrease in river water mass flows and concentrations (especially during first lockdown). Although it is an unappreciative task to speculate about the reasons for this decrease, one might presume that fewer social interactions during lockdowns caused a lower number of stressful situations. Two anti-inflammatory drugs, steroidal dexamethasone and non-steroidal 4-formylaminoantipyrine (a metabolite of aminophenazone), both showed significantly lowered mass flows in Sava River during lockdowns. This is not so much a surprise for the first lockdown since the number of COVID-19 patients did not increase until November 2020, but is puzzling in the case of the second lockdown. In this case, it could be that the increased use of anti-inflammatory drugs in the treatment of difficult COVID-19 patients played only a minor role in the total consumption of these medicaments in the general population. However, when deciphering the influence of lockdowns on river pollution we must consider that the consumption of some pharmaceuticals and personal care products for controlling and preventing COVID-19 increased during the pandemic [11]. In this study, we can see the rise of ATB azithromycin concentrations during the first lockdown (April–June 2020, Supplementary Material) that cannot be attributed to the treatment of COVID-19 as there were too few patients, but probably to seasonal colds, and a sharp increase in the October with the start of the second COVID-19 wave, followed by a decrease during the second lockdown when it seized to be an obligatory part of the COVID-19 therapy. Chen et al. [11] also reported higher detection frequency and concentrations of azithromycin in the aquatic environment of Wuhan, China, during summer 2020 than historically reported. Furthermore, Nason et al. [12] detected elevated concentrations of hydroxychloroquine, PhAC used in the treatment of COVID-19, in sludge from a wastewater-treatment plant (WWTP) in Connecticut, USA, during the initial COVID-19 outbreak and associated lockdown.

Interestingly, an increase in the concentration of cocaine is visible during the two lockdown periods. Such an increase was also observed by Nason et al. [12] in the period from March to June 2020. In the same study, concentrations of the majority of analyzed antidepressants in the WWTP sludge increased, but, like in our case, not venlafaxine. Concentrations of MDMA-3,4 were very low during the first lockdown, but then they increased during the summer period, which was followed by a further dramatical increase during the second lockdown, that was not as strict as the first one. Based on studies conducted on wastewaters, the first COVID-19 lockdown caused decreased use of cocaine, amphetamine, and MDMA-3,4 in Austria, Netherlands, and Spain [31,32]. Moreover, in Italy, an analysis of hair samples from drug users found that cocaine and MDMA use dropped significantly during the initial lockdown (March–May 2020) [33]. These findings on the IDrgs use during first lockdown are complementary with our data on MDMA-3,4 but not on cocaine.

The PCA scores plots revealed several clusters and individual groups according to their occurrence in the river water during the 16 months in 2020 and 2021 that included two major COVID-19 lockdowns. While ATD and DIUR were shown to be affected by both lockdowns, the group containing ATE, AID, and STM showed a more pronounced decrease during the first lockdown. Finally, IDRG and ATB showed a large increase in water mass flows in October 2020 that gradually decreased during the second lockdown, while cardiovascular drugs (CAR) increased during the second lockdown. The results of this study are indicative of lower consumption of some pharmaceuticals that might have caused the lower overall contamination of river waters with organic contaminants, but the causality of this event is difficult to unravel and would require more in-depth analyses and data that are not available at this time. Although this is the only study that analyzed time patterns of group of PhACs/IDrgs in river water over the longer time period (16 months) that encompassed both Covid lockdowns, one must be aware of certain drawbacks of such chemical analyses that are the result of a singular event of water sampling (per month) that could be influenced by multiple factors like the time of the day or the days of the week.

The results of this study confirm that Covid lockdowns caused lower cumulative mass flows of 18 measured PhACs/IDrgs in the Sava and Drava Rivers in Croatia. This was not influenced by the increased use of drugs for the treatment of the COVID-19, like antibiotics and steroidal anti-inflammatory drugs. The decrease of measured PhACs/IDrgs mass flows was more pronounced during the first lockdown, which was stricter than the second one.

## Figures and Tables

**Table 1:** Eighteen chemicals analyzed in this study grouped in eight categories (ATB, ATD, ATE, IDRG, CAR, DIUR, STM, and AID).

CATEGORY	NAME
ANTIBIOTICS (ATB)	Azithromycin
ANTIDEPRESSANTS (ATD)	O-Desmethylvenlafaxine, Venlafaxine
ANTIEPILEPTICS/NEUROLEPTICS (ATE)	10-Hydroxycarbazepine, Amisulpride, Carbamazepine, Lamotrigine
ILLICIT DRUGS (IDRG)	Benzoylecgonine, Cocaine, MDMA-3,4
CARDIOVASCULAR MEDICALS (CAR)	Bisoprolol, Metoprolol
DIURETICS (DIUR)	Torasemide
STIMULANTS (STM)	Caffeine, Cotinine
ANTI-INFLAMMATORY DRUGS (AID)	4-Acetylaminoantipyrine, 4-Formylaminoantipyrine, Dexamethasone

**Table 2:** Loss in PhACs/IDrgs mass flows in Sava River during lockdowns. *—PhACs/IDrgs with F ≥ 1.5.

PhACs/IDrgs	F	StDev	PhACs/IDrgs	F	StDev
Venlafaxine	3.77 *	3.03	Caffein	0.98	0.51
4-Formylaminoantipyrine	2.99 *	1.61	4-Acetylaminoantipyrine	0.91	0.14
O-Desmethylvenlafaxine	2.43 *	1.13	Amisulpride	0.87	0.27
Cotinine	2.34 *	0.74	Cocaine	0.87	0.61
Torasemide	2.00 *	0.98	Azithromycin	0.82	0.31
Dexamethasone	1.65 *	0.94	MDMA-3,4	0.68	0.71
10-Hydroxycarbazepine	1.05	0.29	Carbamazepine	0.51	0.24
Benzoylecgonine	1.03	0.17	Bisoprolol	0.25	0.10
Lamotrigine	1.02	0.28	Metoprolol	0.23	0.15

**Table 3:** Loss in PhACs/IDrgs mass flows in Drava River during lockdowns. *—PhACs/IDrgs with F ≥ 1.5.

PhACs/IDrgs	F	StDev
Cotinine	7.11 *	6.36
Caffein	2.21 *	0.54
Lamotrigine	1.93 *	0.65
Venlafaxine	1.70 *	0.24
O-Desmethylvenlafaxine	1.48	0.60
10-Hydroxycarbazepine	1.03	0.34
Carbamazepine	0.66	0.08

## Data Availability

Not applicable.

## Supplementary Materials

The following supporting information can be downloaded at https://www.mdpi.com/article/10.3390/toxics10050241/s1: Figure S1, PhACs/IDrgs concentrations in Sava River; Figure S2, PhACs/IDrgs concentrations in Drava River; Table S1, PhACs/IDrgs concentrations in Sava and Drava River; Table S2, PhACs/IDrgs water flows in Sava and Drava River.
