# Supplementary material for: COVID-19 Lockdowns—Effect on Concentration of Pharmaceuticals and Illicit Drugs in Two Major Croatian Rivers
Source: Toxics. 2022 May 10;10(5):241. doi: 10.3390/toxics10050241 (PMC9143423; doi:10.3390/toxics10050241)
Supplement: Supplementary file 1 [file toxics-10-00241-s001.zip › toxics-1696703-supplementary figures.pdf]

COVID-19 Lockdowns—Effect on Concentration of Pharmaceuticals and Illicit Drugs in Two Major Croatian Rivers

Draženka Stipaničev, Siniša Repec, Matej Vucić, Mario Lovrić and Göran Klobučar

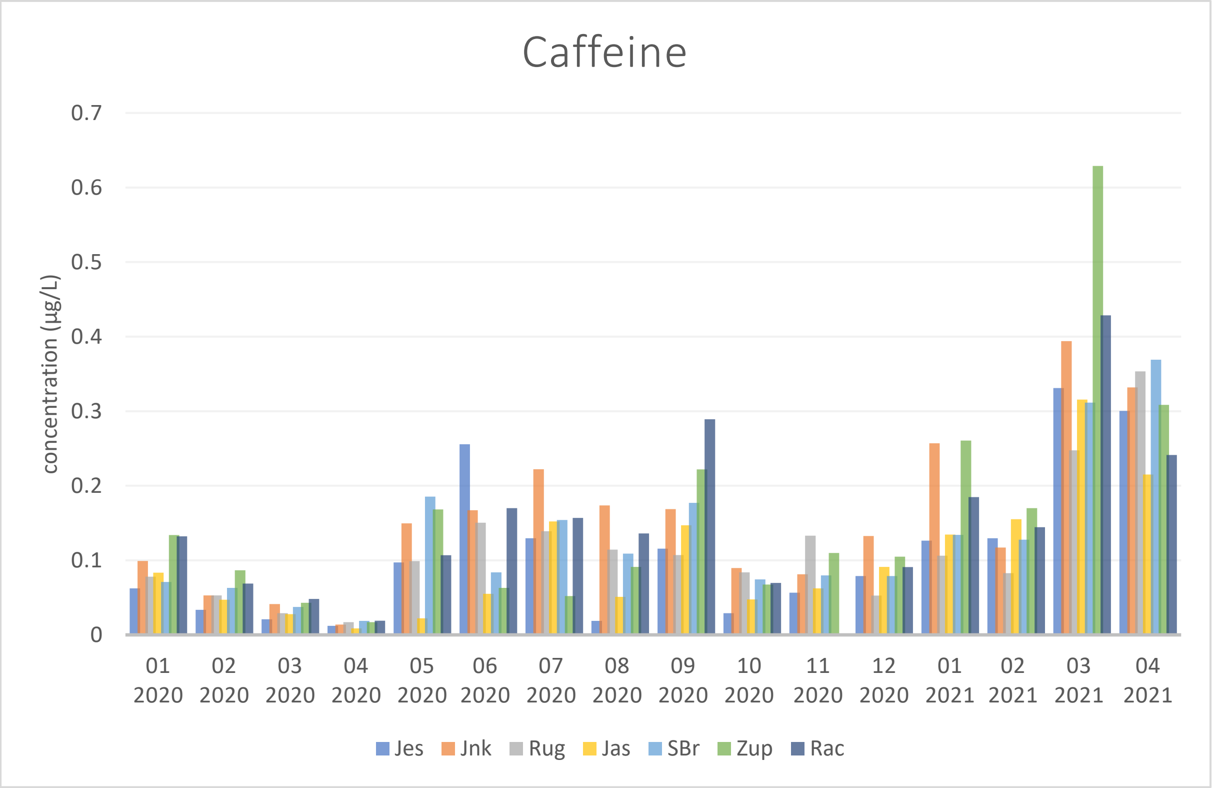

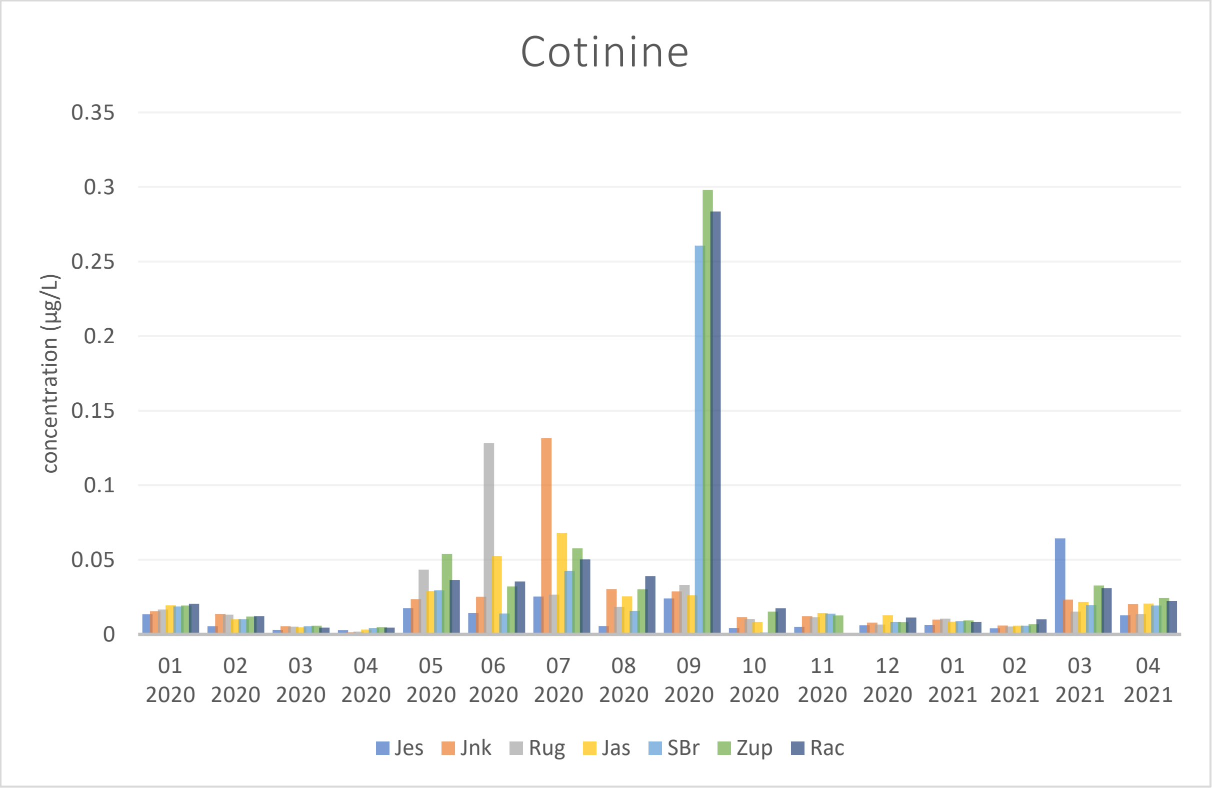

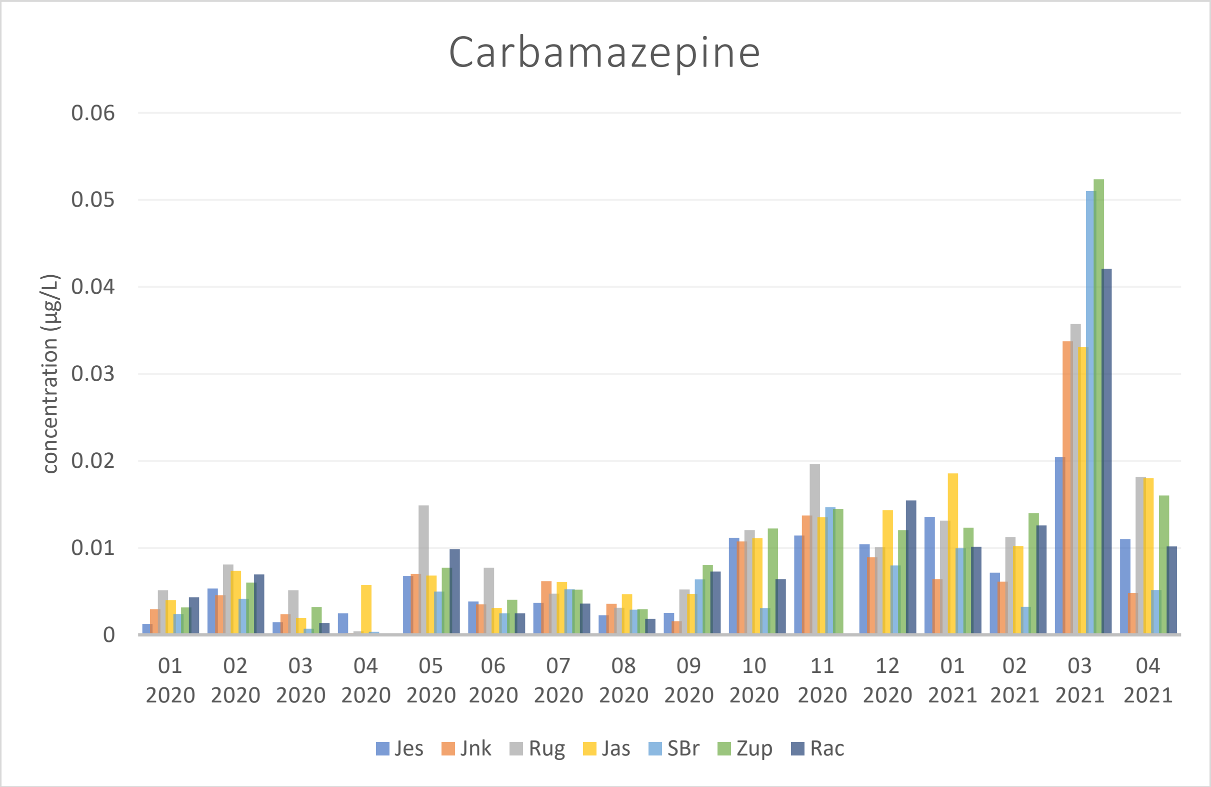
Carbamazepine
concentration (µg/L)
0.06
0.05
0.04
0.03
0.02
0.01
0
01 2020
02 2020
03 2020
04 2020
05 2020
06 2020
07 2020
08 2020
09 2020
10 2020
11 2020
12 2020
01 2021
02 2021
03 2021
04 2021
Jes Jnk Rug Jas SBr Zup Rac

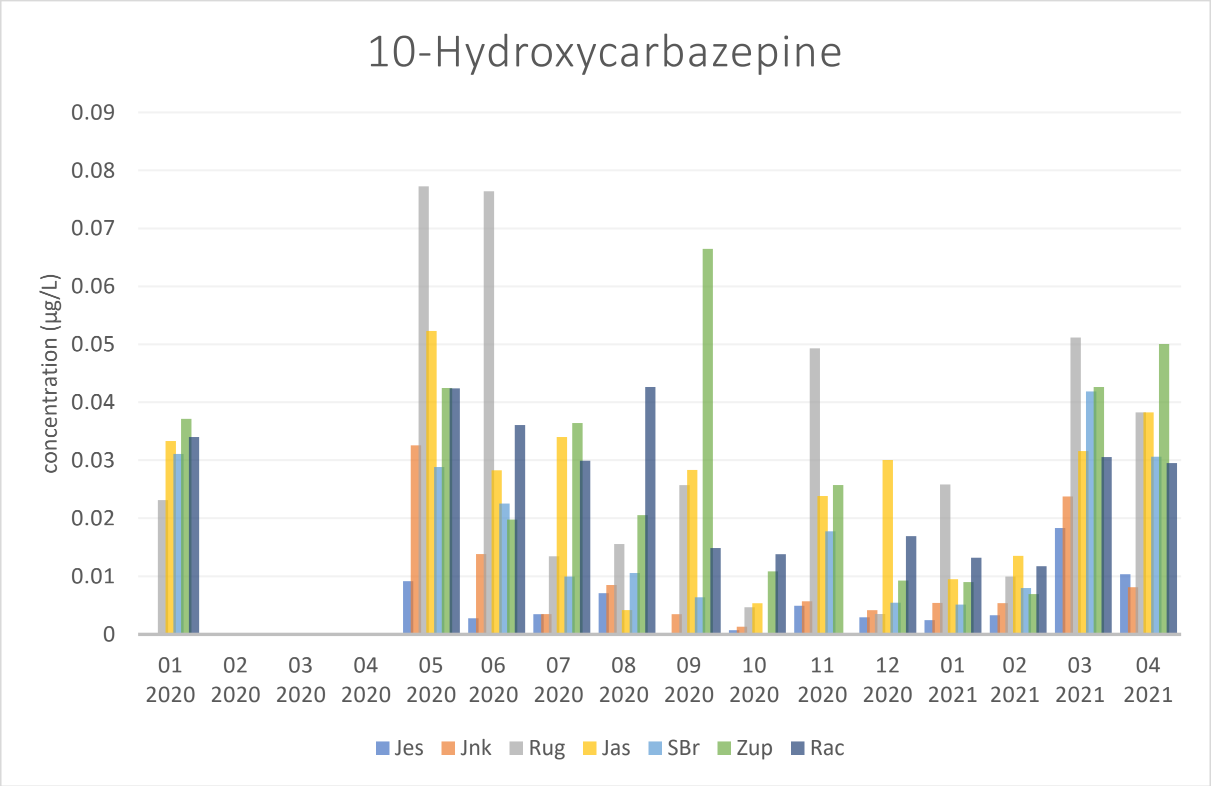
10-Hydroxycarbazepine
concentration (µg/L)
0.09
0.08
0.07
0.06
0.05
0.04
0.03
0.02
0.01
0
01 2020
02 2020
03 2020
04 2020
05 2020
06 2020
07 2020
08 2020
09 2020
10 2020
11 2020
12 2020
01 2021
02 2021
03 2021
04 2021
Jes Jnk Rug Jas SBr Zup Rac

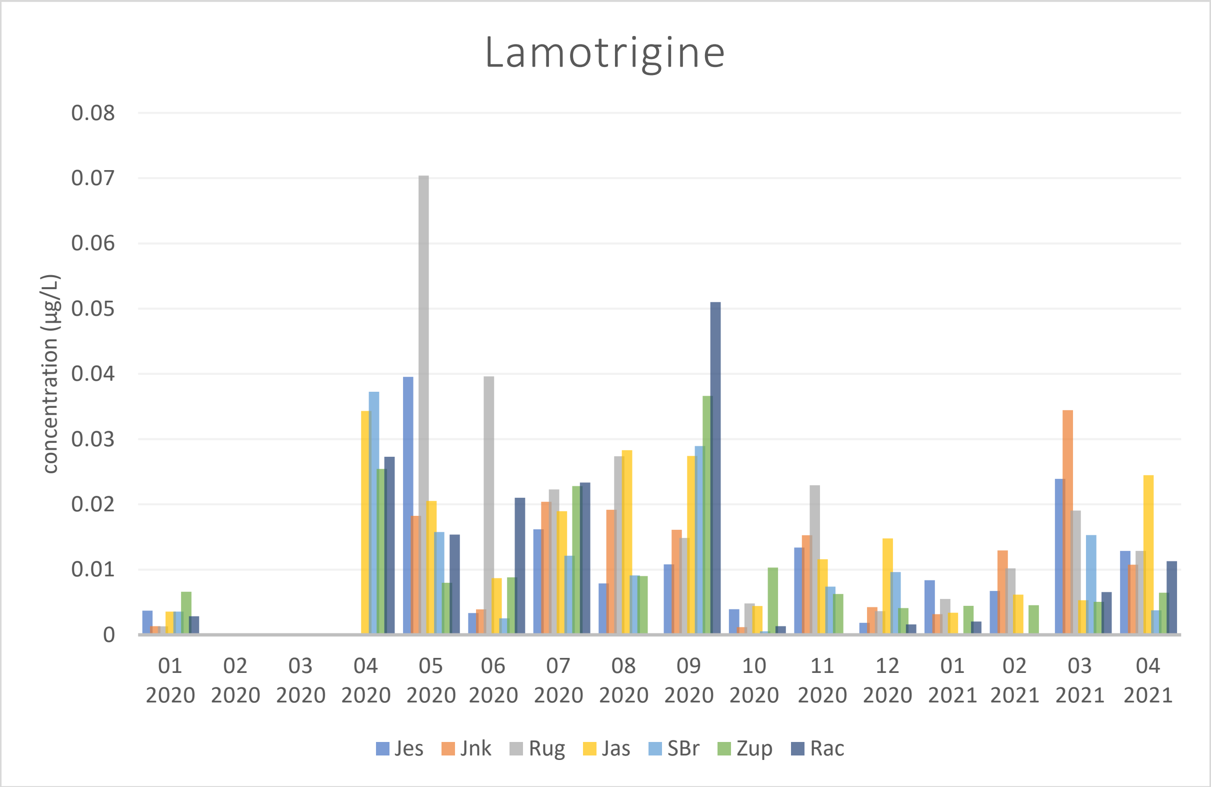

Lamotrigine
0.08
0.07
0.06
0.05
0.04
0.03
0.02
0.01
0
concentration (µg/L)
01 2020
02 2020
03 2020
04 2020
05 2020
06 2020
07 2020
08 2020
09 2020
10 2020
11 2020
12 2020
01 2021
02 2021
03 2021
04 2021
Jes
Jnk
Rug
Jas
SBr
Zup
Rac

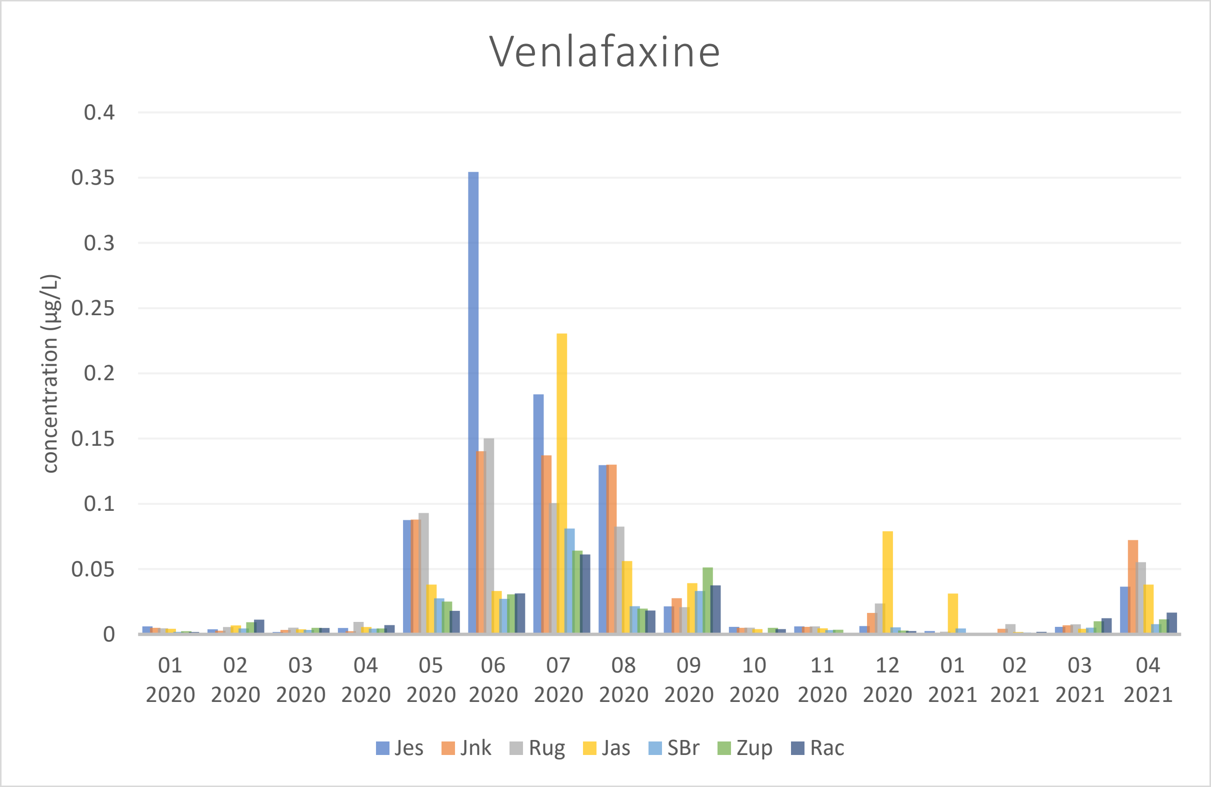

Venlafaxine
0.4
0.35
0.3
0.25
0.2
0.15
0.1
0.05
0
concentration (µg/L)
01 2020
02 2020
03 2020
04 2020
05 2020
06 2020
07 2020
08 2020
09 2020
10 2020
11 2020
12 2020
01 2021
02 2021
03 2021
04 2021
Jes
Jnk
Rug
Jas
SBr
Zup
Rac

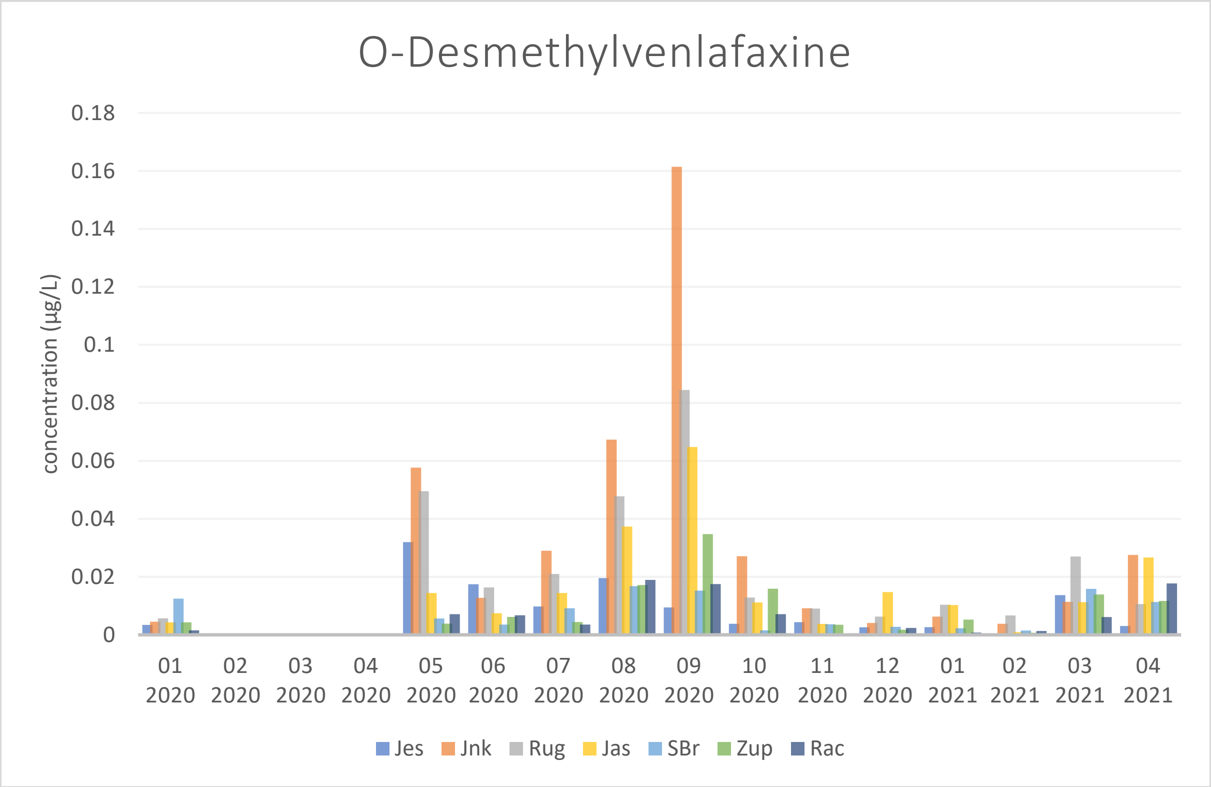

O-Desmethylvenlafaxine
concentration (µg/L)
0.18
0.16
0.14
0.12
0.1
0.08
0.06
0.04
0.02
0
01 2020
02 2020
03 2020
04 2020
05 2020
06 2020
07 2020
08 2020
09 2020
10 2020
11 2020
12 2020
01 2021
02 2021
03 2021
04 2021
Jes
Jnk
Rug
Jas
SBr
Zup
Rac

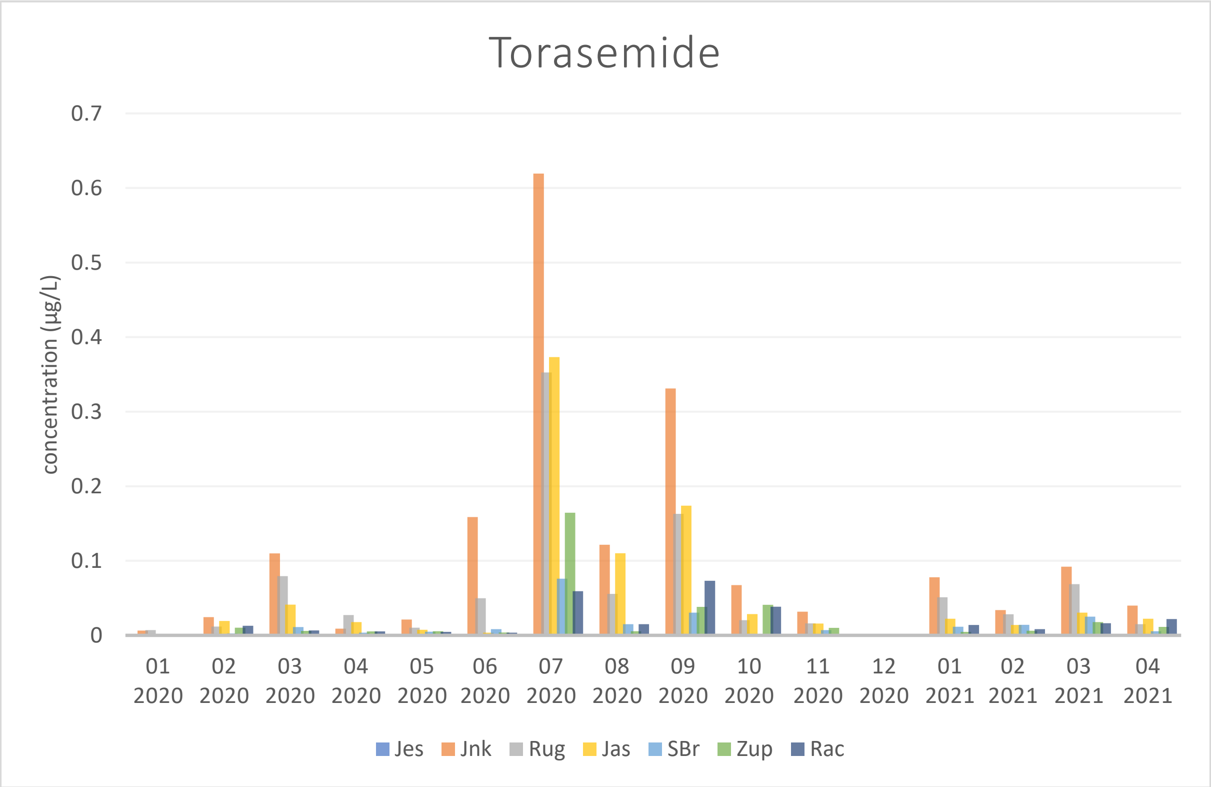

Torasemide
concentration (µg/L)
0.7
0.6
0.5
0.4
0.3
0.2
0.1
0
01 2020
02 2020
03 2020
04 2020
05 2020
06 2020
07 2020
08 2020
09 2020
10 2020
11 2020
12 2020
01 2021
02 2021
03 2021
04 2021
Jes
Jnk
Rug
Jas
SBr
Zup
Rac

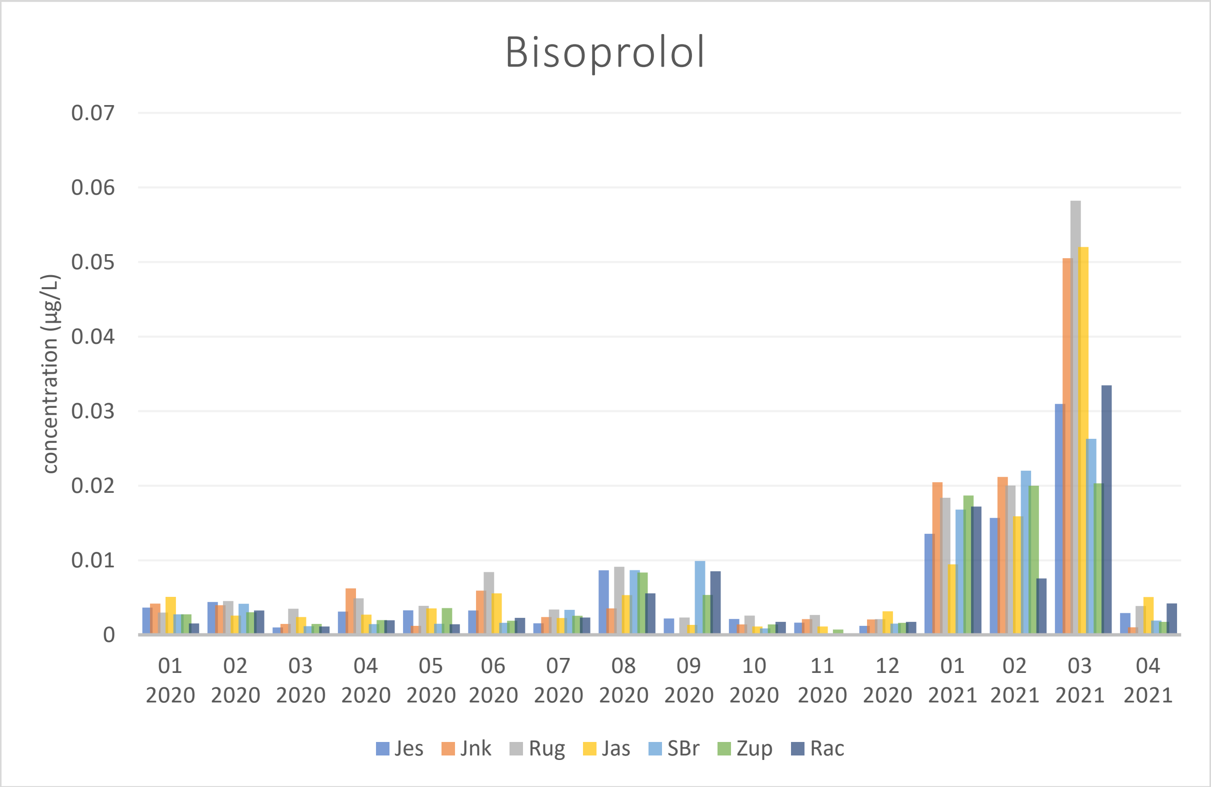
Bisoprolol
concentration (µg/L)
0.07
0.06
0.05
0.04
0.03
0.02
0.01
0
01 2020
02 2020
03 2020
04 2020
05 2020
06 2020
07 2020
08 2020
09 2020
10 2020
11 2020
12 2020
01 2021
02 2021
03 2021
04 2021
Jes
Jnk
Rug
Jas
SBr
Zup
Rac

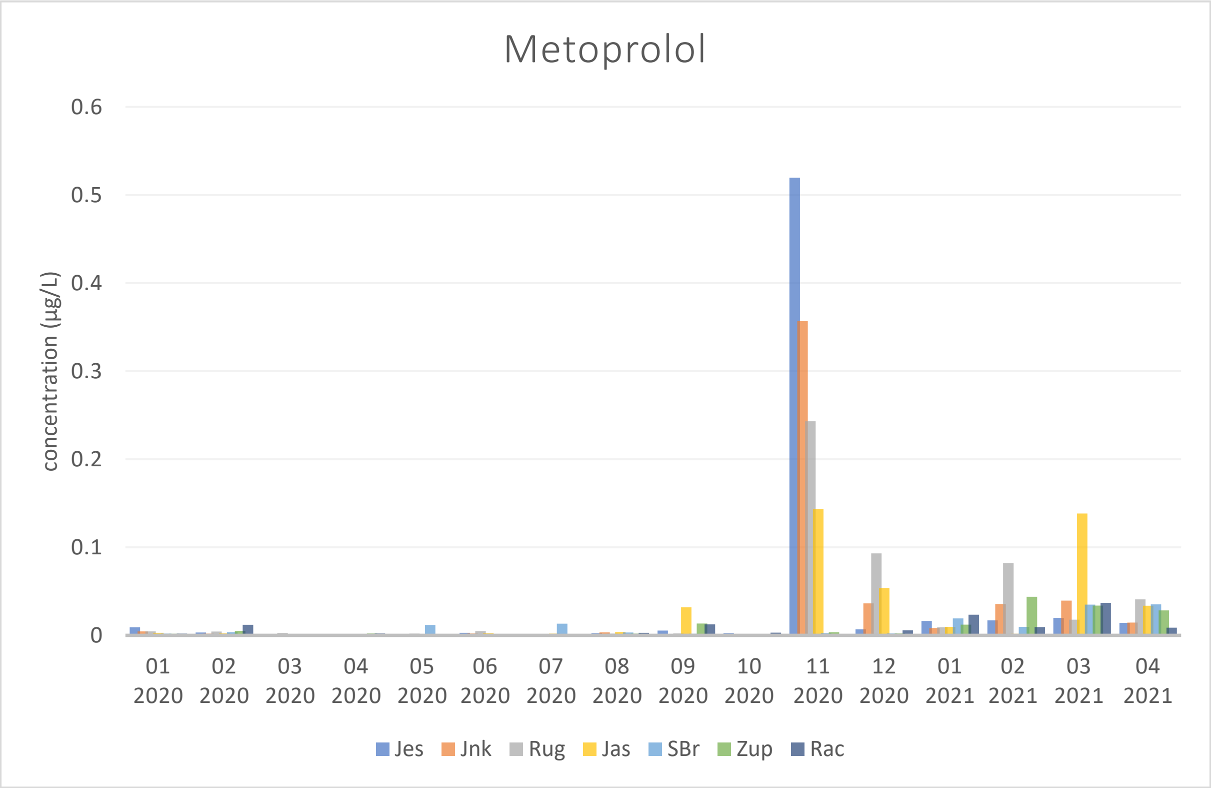
Metoprolol
concentration (µg/L)
0.6
0.5
0.4
0.3
0.2
0.1
0
01 2020
02 2020
03 2020
04 2020
05 2020
06 2020
07 2020
08 2020
09 2020
10 2020
11 2020
12 2020
01 2021
02 2021
03 2021
04 2021
Jes
Jnk
Rug
Jas
SBr
Zup
Rac

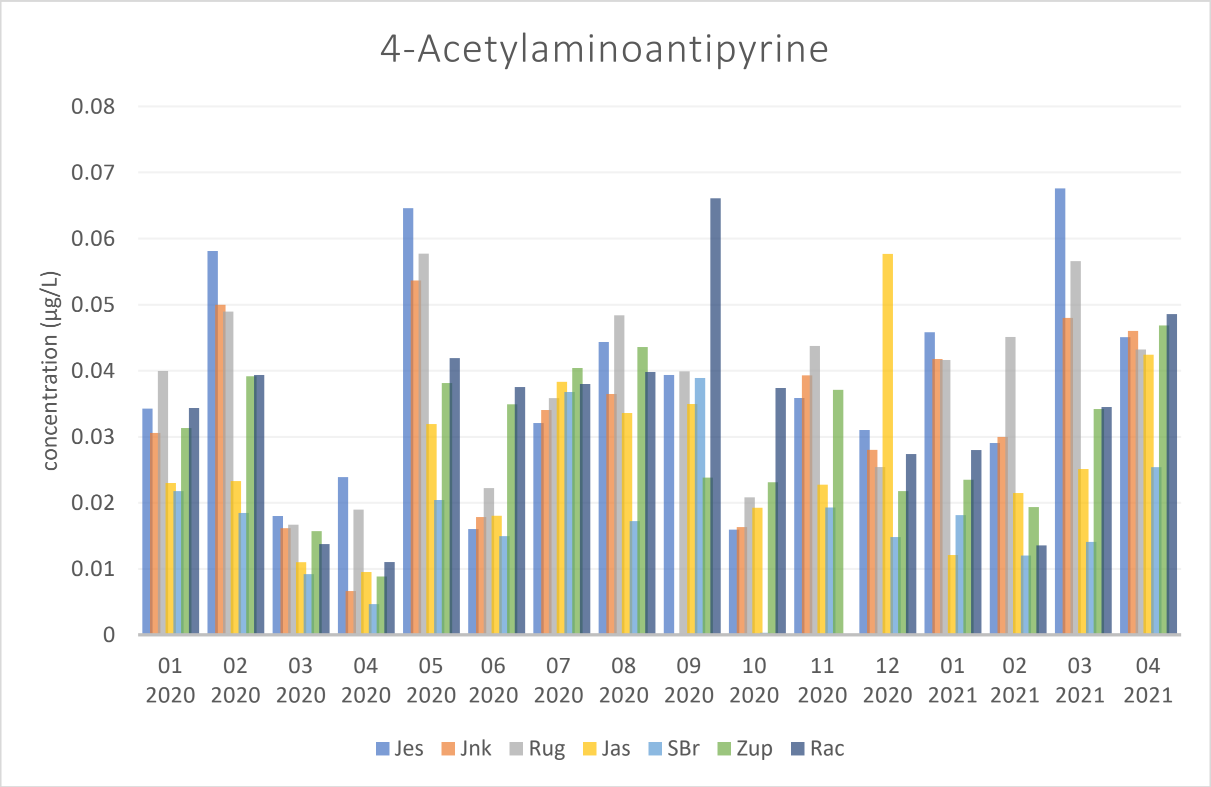
4-Acetylaminoantipyrine
concentration (µg/L)
0.08
0.07
0.06
0.05
0.04
0.03
0.02
0.01
0
01 2020
02 2020
03 2020
04 2020
05 2020
06 2020
07 2020
08 2020
09 2020
10 2020
11 2020
12 2020
01 2021
02 2021
03 2021
04 2021
Jes
Jnk
Rug
Jas
SBr
Zup
Rac

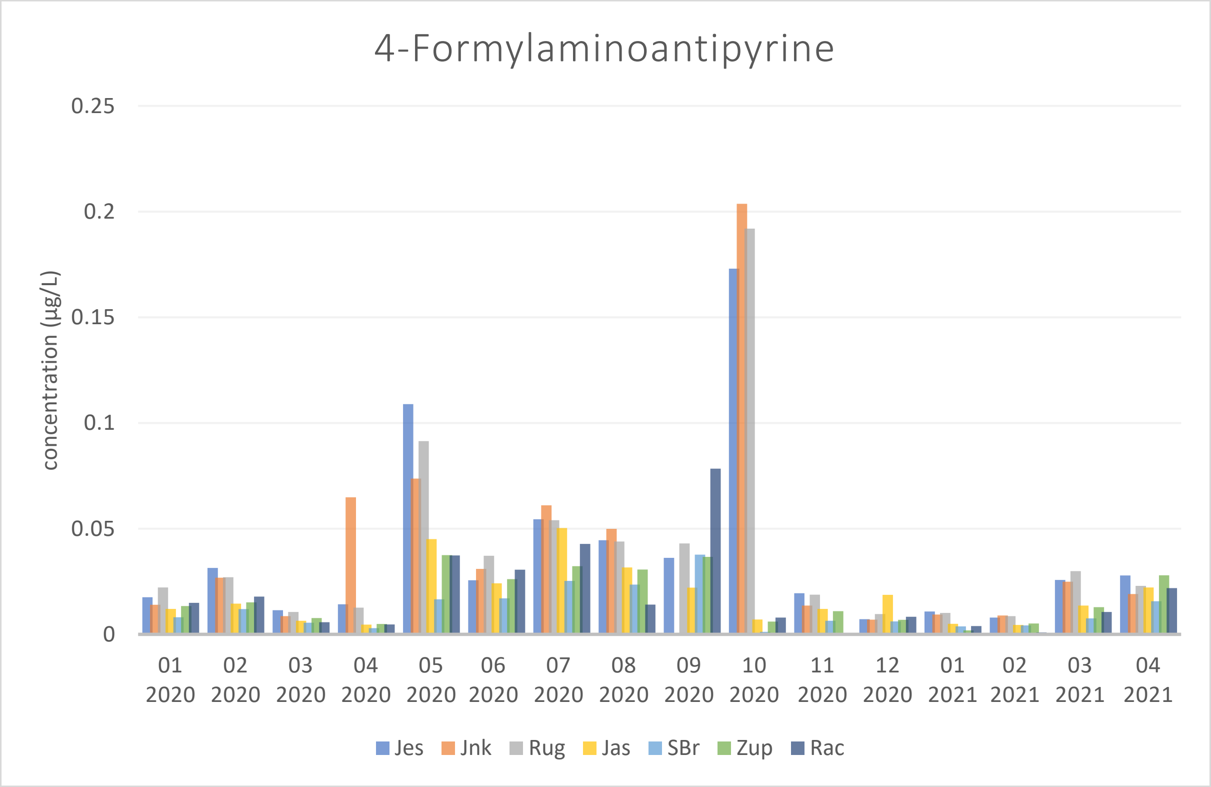
4-Formylaminoantipyrine
concentration (µg/L)
0.25
0.2
0.15
0.1
0.05
0
01 2020
02 2020
03 2020
04 2020
05 2020
06 2020
07 2020
08 2020
09 2020
10 2020
11 2020
12 2020
01 2021
02 2021
03 2021
04 2021
Jes
Jnk
Rug
Jas
SBr
Zup
Rac

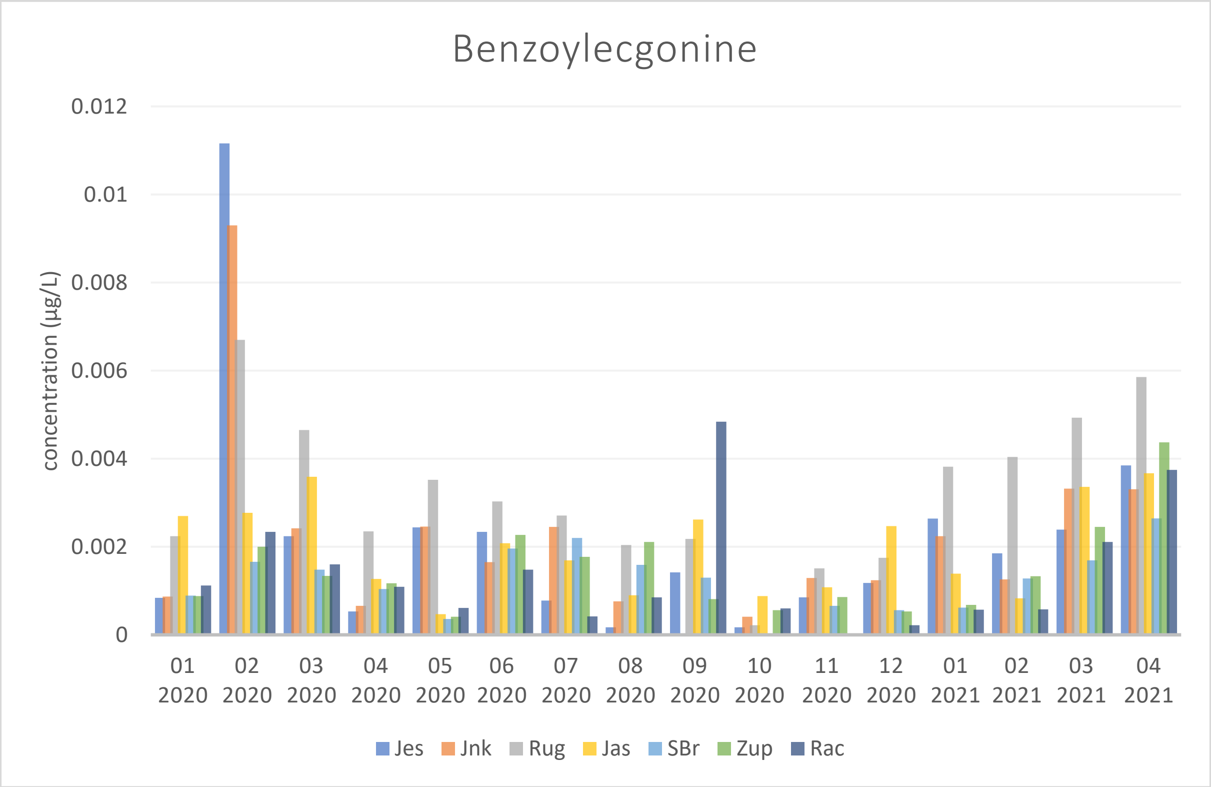
Benzoylecgonine
concentration (µg/L)
0.012
0.01
0.008
0.006
0.004
0.002
0
01 2020
02 2020
03 2020
04 2020
05 2020
06 2020
07 2020
08 2020
09 2020
10 2020
11 2020
12 2020
01 2021
02 2021
03 2021
04 2021
Jes Jnk Rug Jas SBr Zup Rac

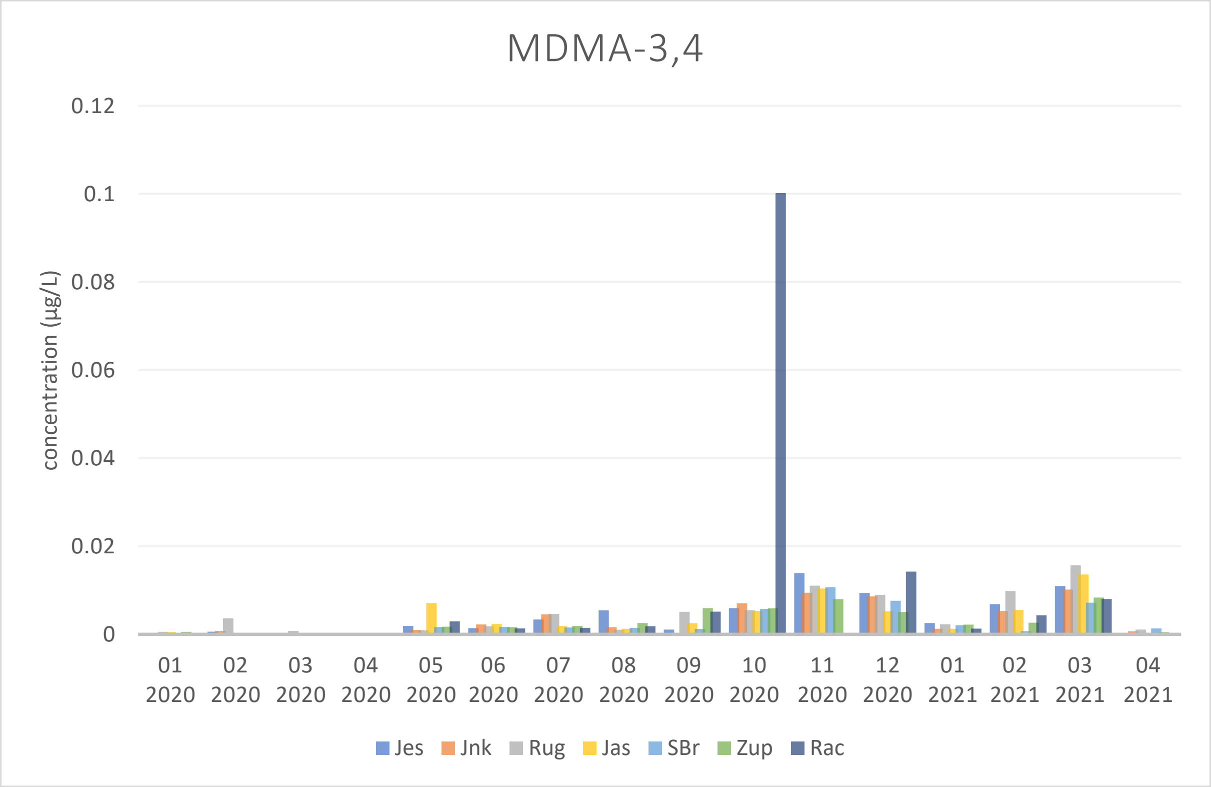
MDMA-3,4
concentration (µg/L)
0.12
0.1
0.08
0.06
0.04
0.02
0
01 2020
02 2020
03 2020
04 2020
05 2020
06 2020
07 2020
08 2020
09 2020
10 2020
11 2020
12 2020
01 2021
02 2021
03 2021
04 2021
Jes Jnk Rug Jas SBr Zup Rac

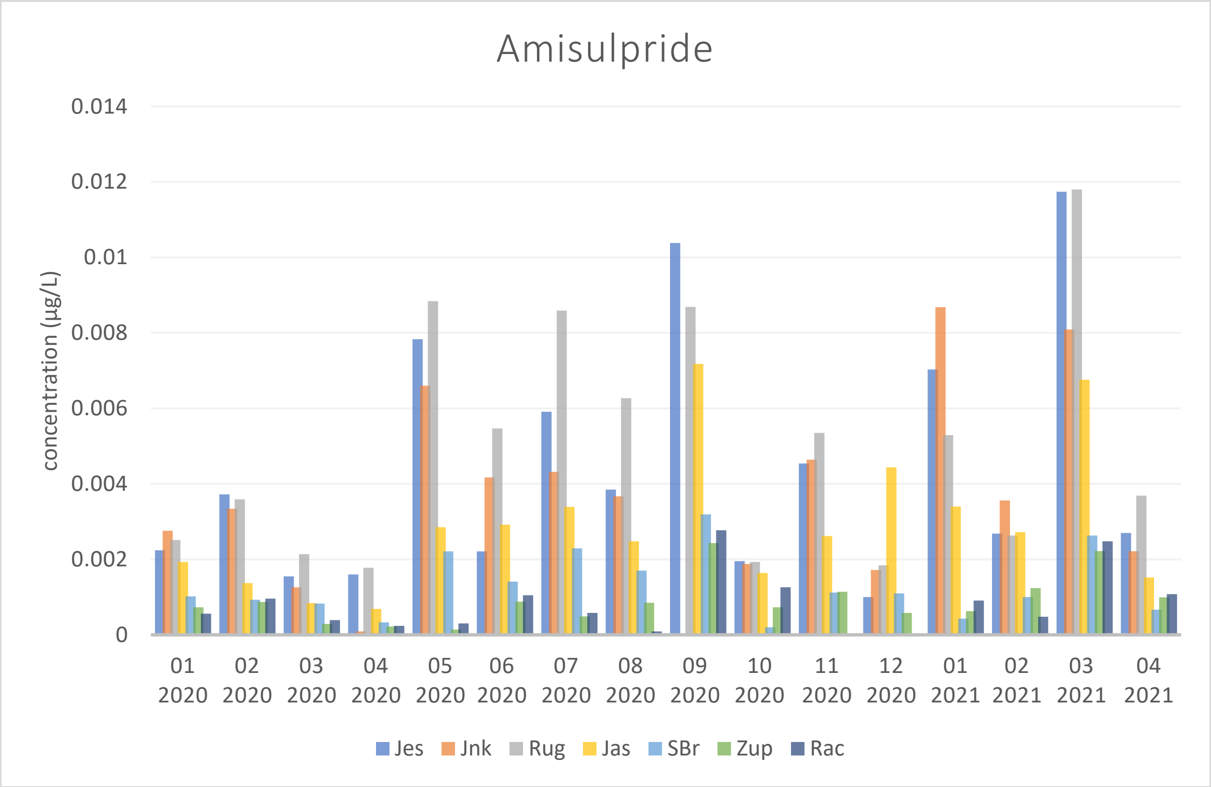
Amisulpride
0.014
0.012
0.01
0.008
0.006
0.004
0.002
0
concentration (µg/L)
01 2020
02 2020
03 2020
04 2020
05 2020
06 2020
07 2020
08 2020
09 2020
10 2020
11 2020
12 2020
01 2021
02 2021
03 2021
04 2021
Jes Jnk Rug Jas SBr Zup Rac

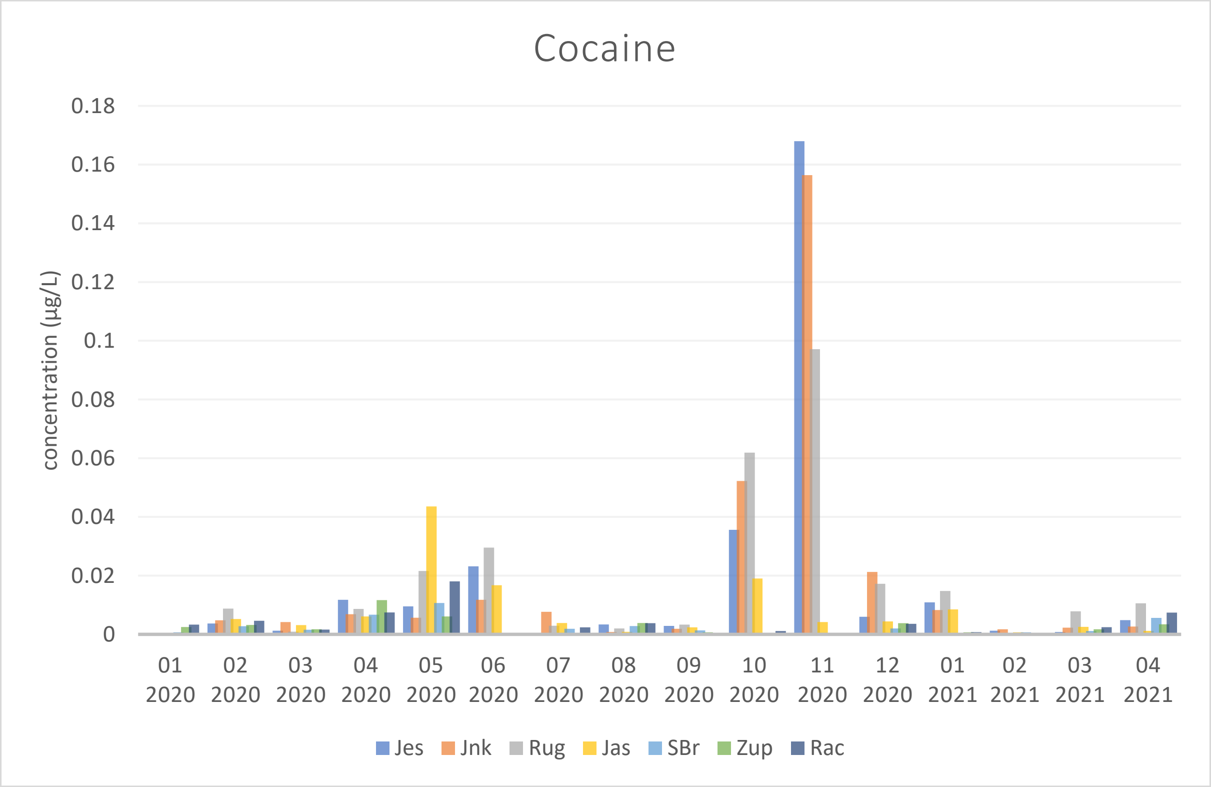
Cocaine
0.18
0.16
0.14
0.12
0.1
0.08
0.06
0.04
0.02
0
concentration (µg/L)
01 2020
02 2020
03 2020
04 2020
05 2020
06 2020
07 2020
08 2020
09 2020
10 2020
11 2020
12 2020
01 2021
02 2021
03 2021
04 2021
Jes Jnk Rug Jas SBr Zup Rac

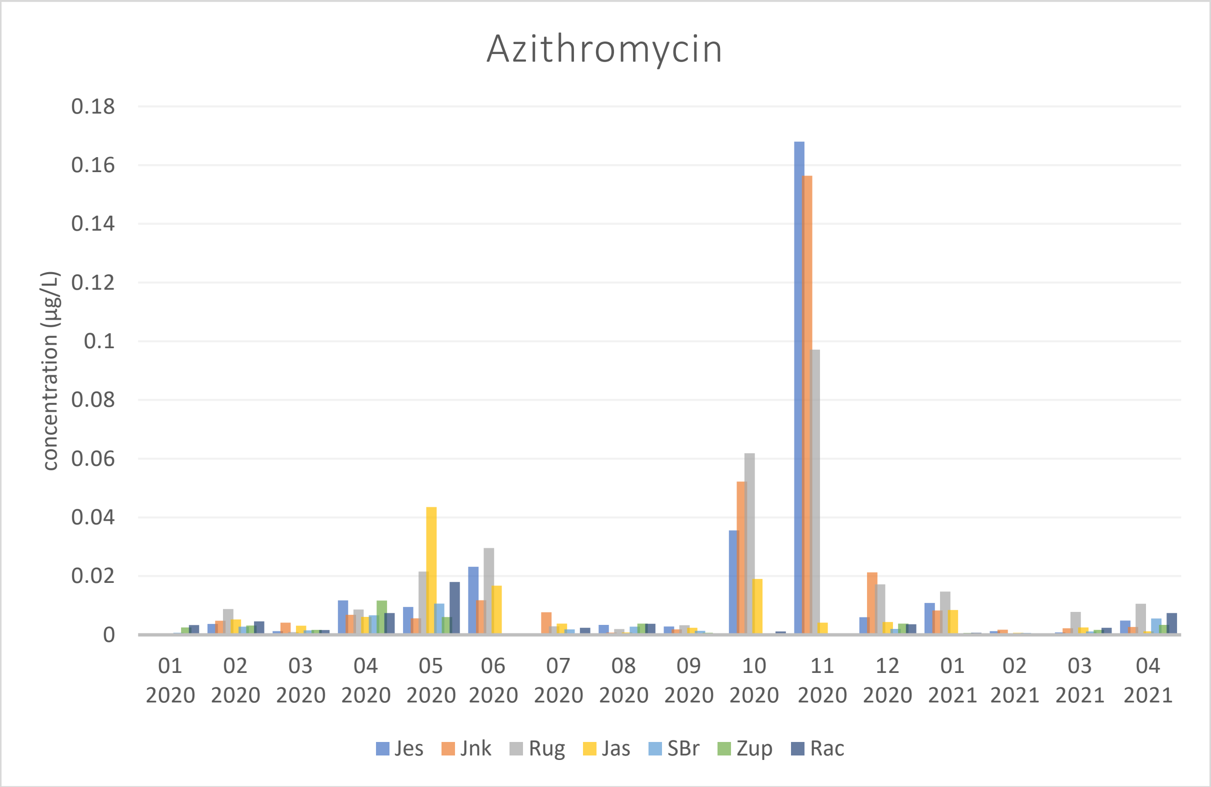

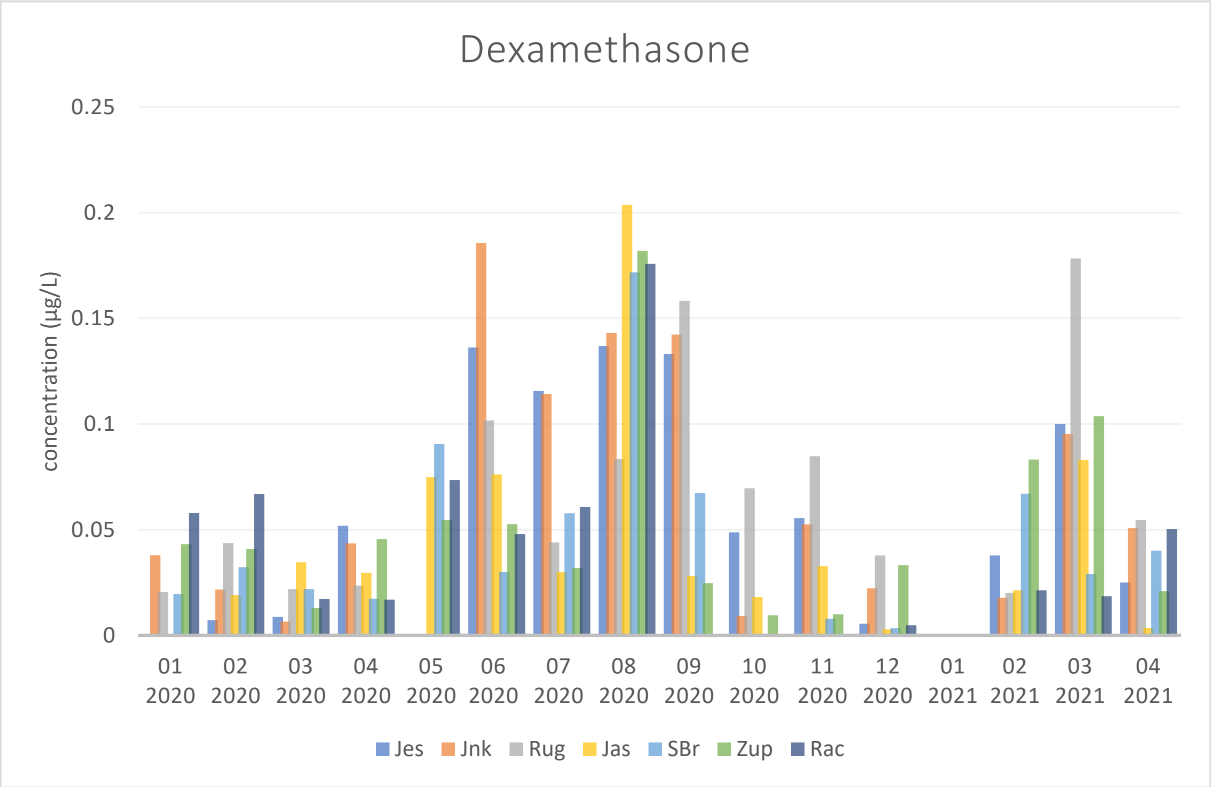

Figure S1. PhACs/IDrgs concentrations in Sava River.

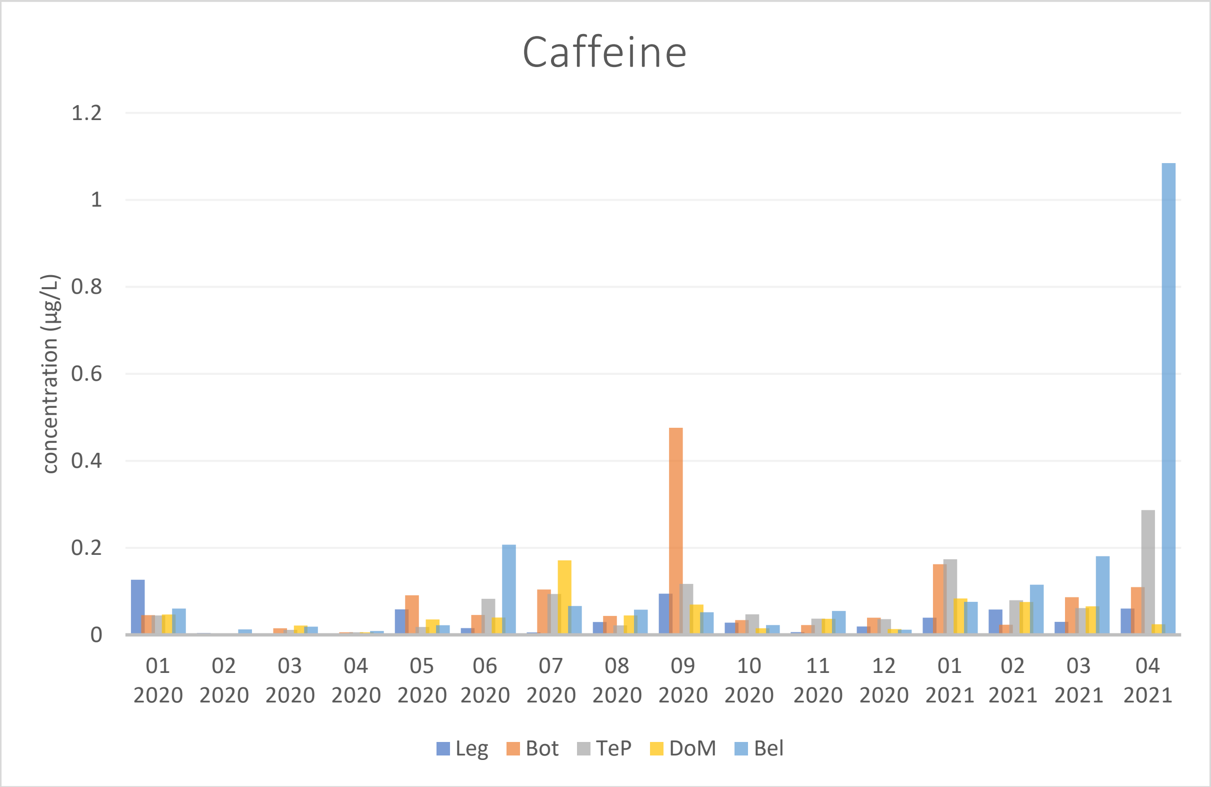
Caffeine
concentration (µg/L)
1.2
1
0.8
0.6
0.4
0.2
0
01 2020
02 2020
03 2020
04 2020
05 2020
06 2020
07 2020
08 2020
09 2020
10 2020
11 2020
12 2020
01 2021
02 2021
03 2021
04 2021
Leg
Bot
TeP
DoM
Bel

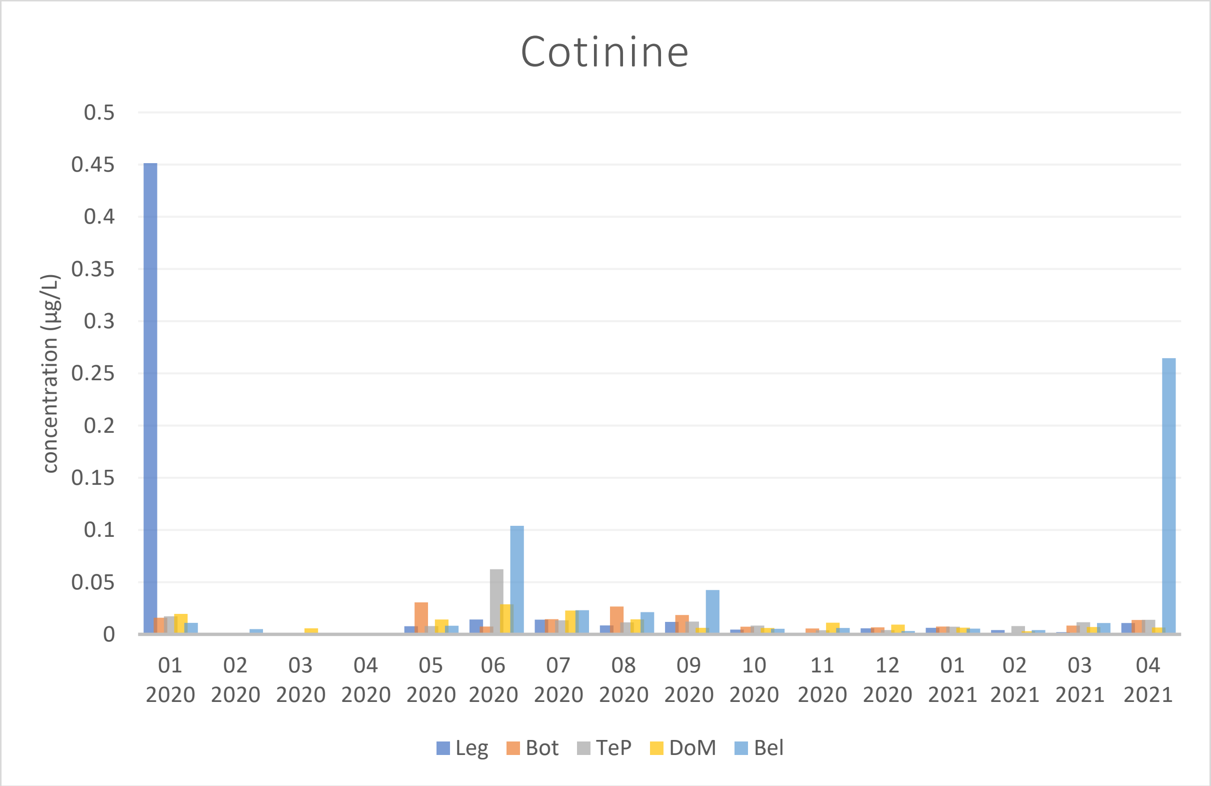
Cotinine
concentration (µg/L)
0.5
0.45
0.4
0.35
0.3
0.25
0.2
0.15
0.1
0.05
0
01 2020
02 2020
03 2020
04 2020
05 2020
06 2020
07 2020
08 2020
09 2020
10 2020
11 2020
12 2020
01 2021
02 2021
03 2021
04 2021
Leg
Bot
TeP
DoM
Bel

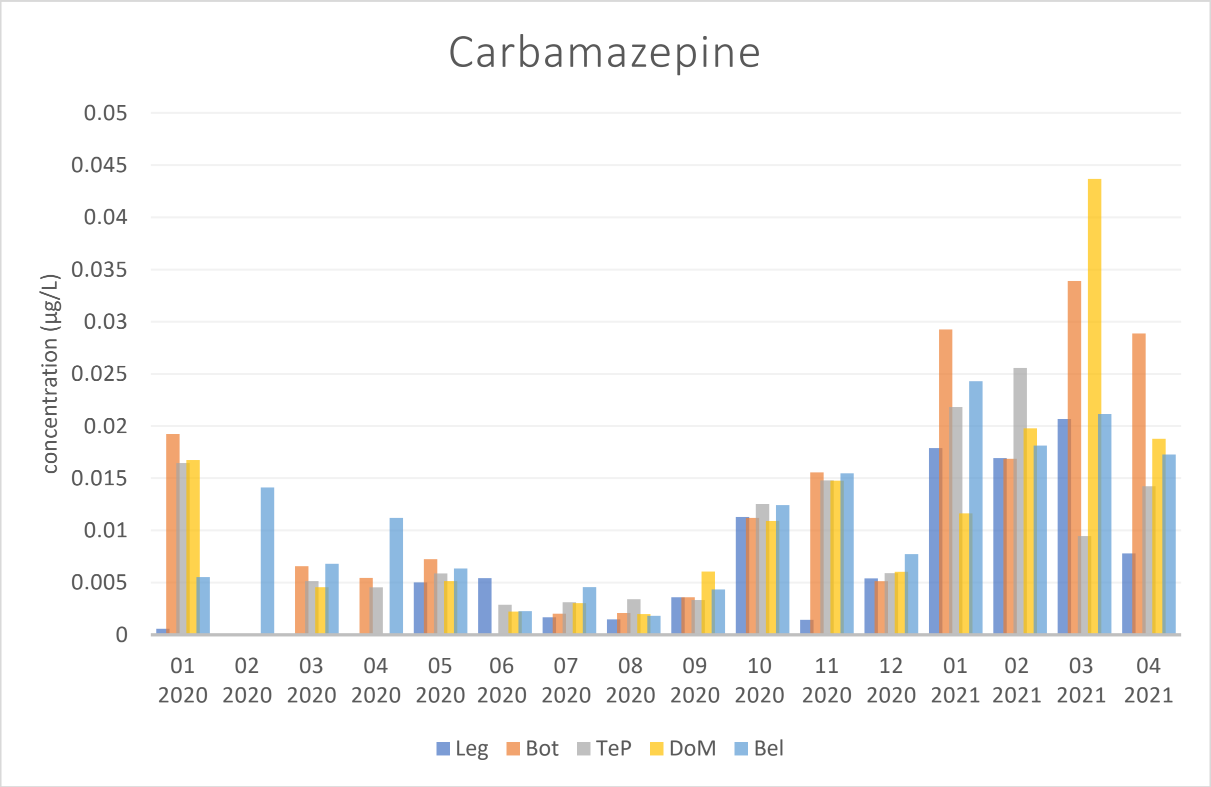
Carbamazepine
0.05
0.045
0.04
0.035
0.03
0.025
0.02
0.015
0.01
0.005
0
concentration (µg/L)
01 2020
02 2020
03 2020
04 2020
05 2020
06 2020
07 2020
08 2020
09 2020
10 2020
11 2020
12 2020
01 2021
02 2021
03 2021
04 2021
Leg
Bot
TeP
DoM
Bel

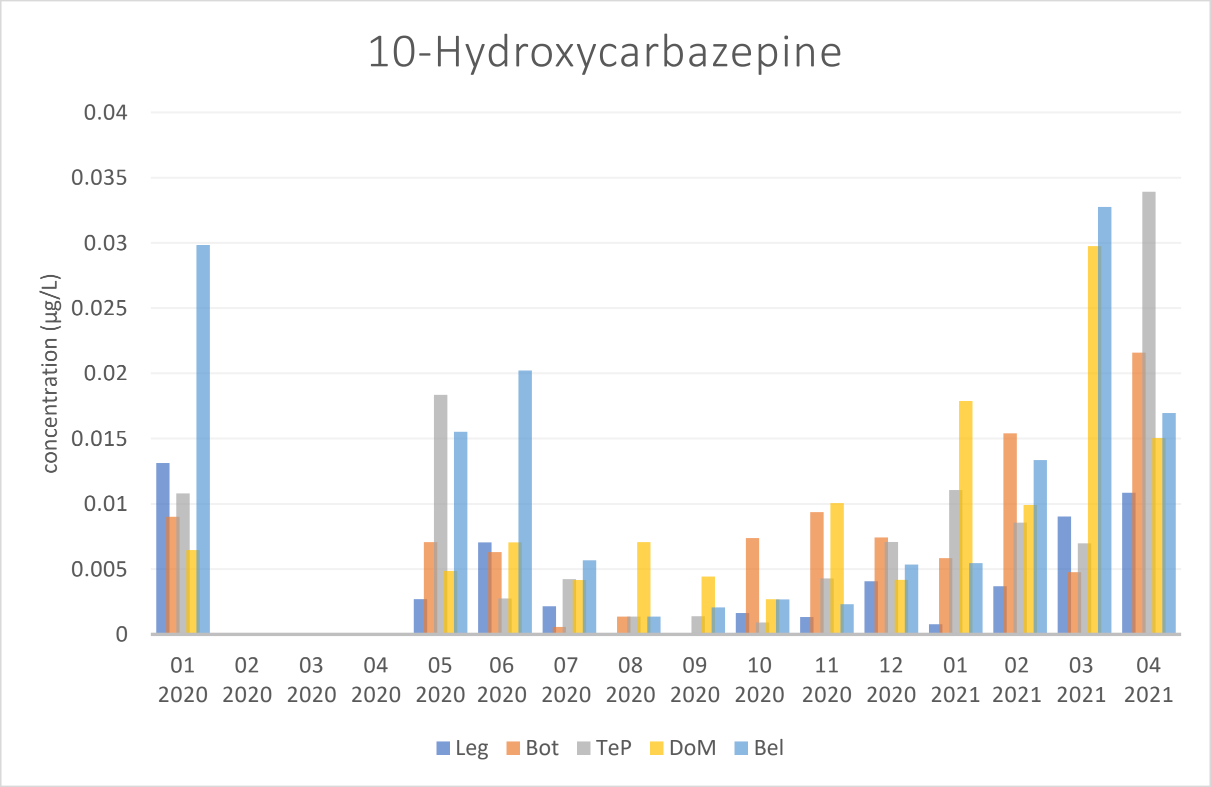
10-Hydroxycarbazepine
0.04
0.035
0.03
0.025
0.02
0.015
0.01
0.005
0
concentration (µg/L)
01 2020
02 2020
03 2020
04 2020
05 2020
06 2020
07 2020
08 2020
09 2020
10 2020
11 2020
12 2020
01 2021
02 2021
03 2021
04 2021
Leg
Bot
TeP
DoM
Bel

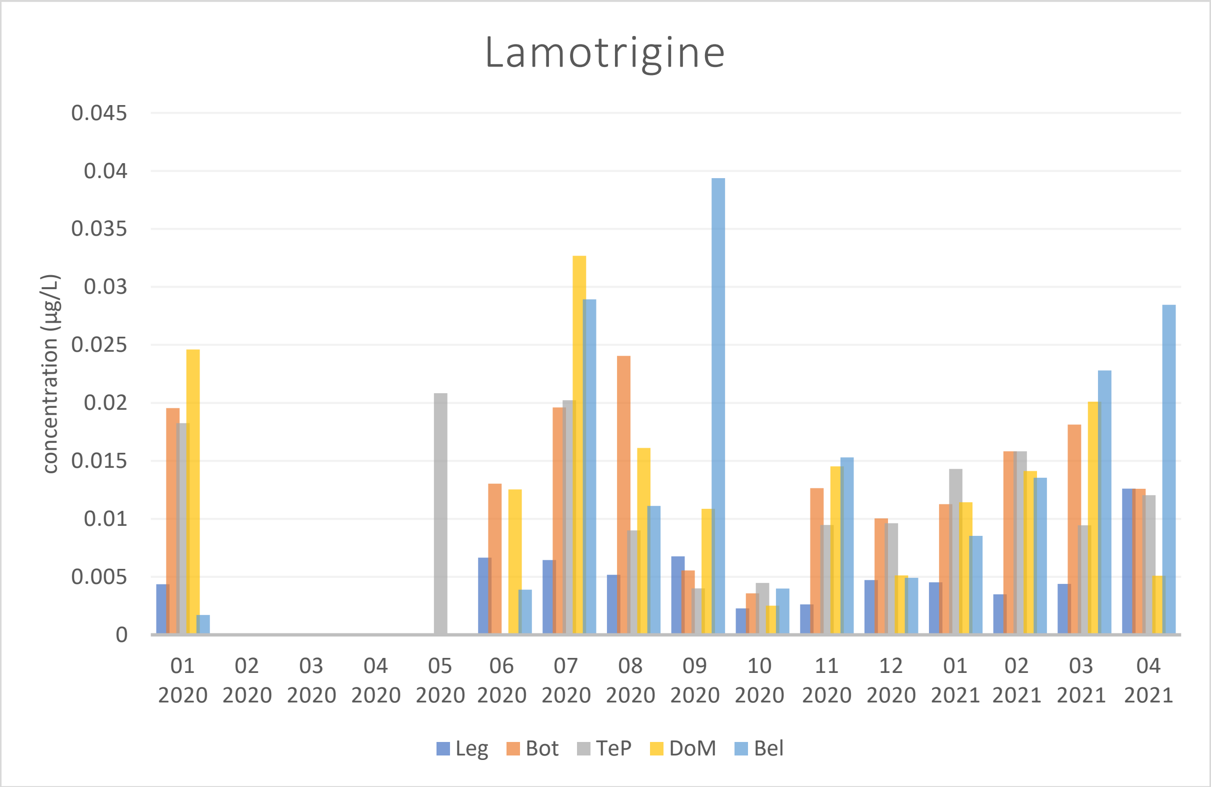
Lamotrigine
concentration (µg/L)
0.045
0.04
0.035
0.03
0.025
0.02
0.015
0.01
0.005
0
01 2020
02 2020
03 2020
04 2020
05 2020
06 2020
07 2020
08 2020
09 2020
10 2020
11 2020
12 2020
01 2021
02 2021
03 2021
04 2021
Leg
Bot
TeP
DoM
Bel

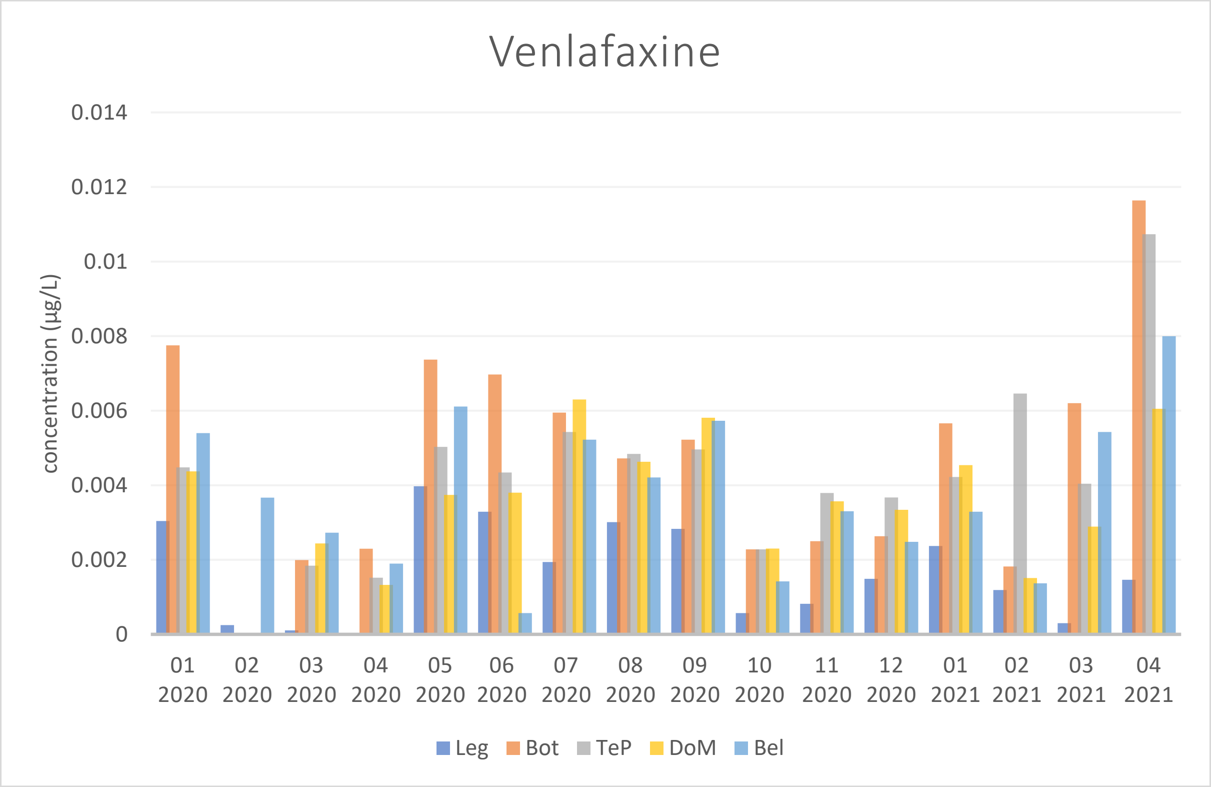
Venlafaxine
concentration (µg/L)
0.014
0.012
0.01
0.008
0.006
0.004
0.002
0
01 2020
02 2020
03 2020
04 2020
05 2020
06 2020
07 2020
08 2020
09 2020
10 2020
11 2020
12 2020
01 2021
02 2021
03 2021
04 2021
Leg
Bot
TeP
DoM
Bel

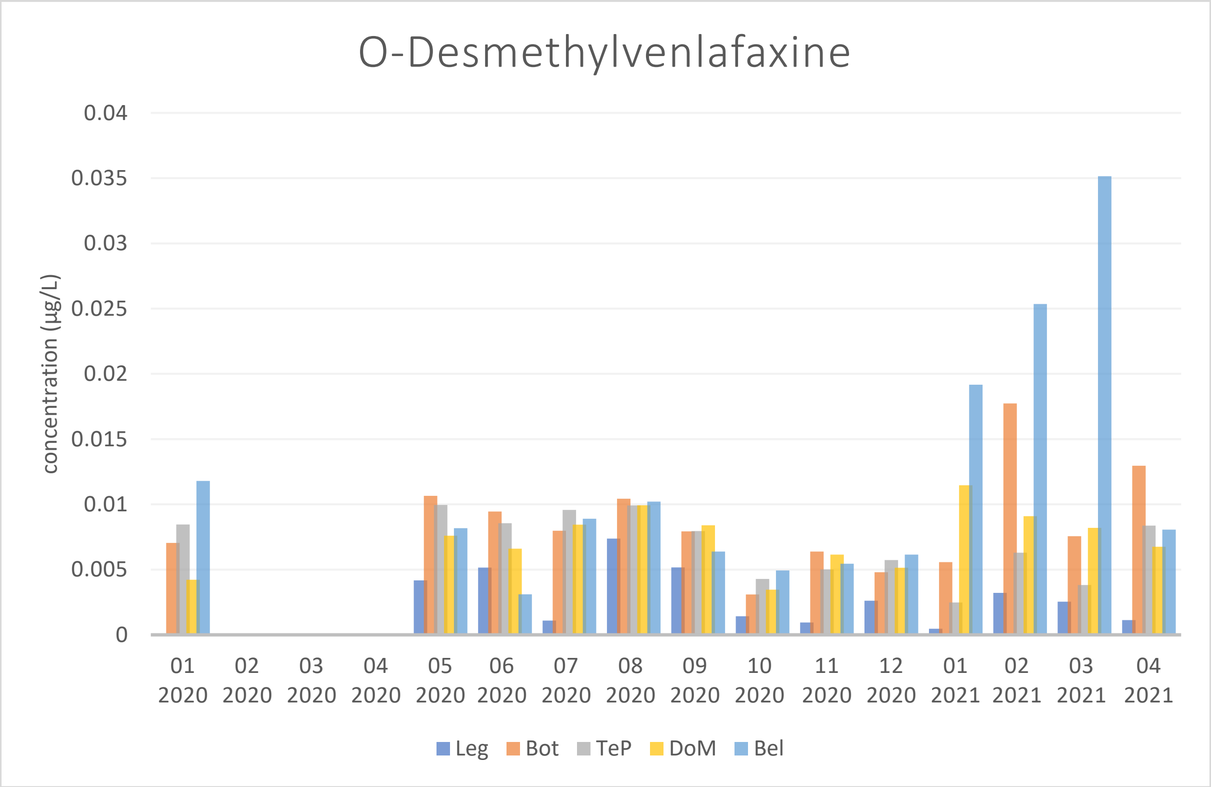

Figure S2. PhACs/IDrgs concentrations in Drava River.
